# Prompt Engineering Accelerates the Data‐Driven Discovery of Photocatalysts via an LLM‐Based Model Ensemble Strategy

**DOI:** 10.1002/advs.202524215

**Published:** 2026-02-16

**Authors:** Dianyuan Li, Xichen Sun, Shaohua Sun, Runzhou Wang, Miaomiao Zhang, Meng Xiao, Yue Wang, Yuezhou Zhang

**Affiliations:** ^1^ Frontiers Science Centre for Flexible Electronics (FSCFE), MIIT Key Laboratory of Flexible Electronics (KLoFE), Shaanxi Key Laboratory of Flexible Electronics, Xi'an Key Laboratory of Flexible Electronics, Xi'an Key Laboratory of Biomedical Materials & Engineering, Xi'an Institute of Flexible Electronics, Institute of Flexible Electronics (IFE) Northwestern Polytechnical University Xi'an Shaanxi China; ^2^ School of Economics and Management Xi'an University of Posts and Telecommunications Xi'an Shaanxi China; ^3^ Key laboratory of Flexible Electronics of Zhejiang Province Ningbo Institute of Northwestern Polytechnical University Ningbo China

**Keywords:** graphitic carbon nitride, large language model, machine learning, model ensemble, prompt engineering

## Abstract

The overwhelming volume of unstructured scientific literature presents a fundamental bottleneck to materials discovery, where critical data on synthesis and properties remain locked in text. Here, a closed‐loop framework that integrates automated knowledge extraction with interpretable machine learning and targeted experimental validation is presented. This approach is centered on a novel data extraction pipeline, which combines a prompt‐engineered large language model with a model ensemble strategy, systematically optimized to interpret complex materials science narratives. When deployed to construct a database for defect‐engineered carbon nitride photocatalysts, the system achieved 90% accuracy and recall for key parameters. Analysis of the high‐fidelity dataset enabled reliable machine learning models to identify specific surface area (170 m^2^ g^−1^) and bandgap (≈2.31 eV) as dominant performance parameters. Crucially, SHapley Additive exPlanations analysis elucidated a non‐monotonic relationship for bandgap, identifying an optimal range of 2.2–2.4 eV and quantifying the fundamental trade‐off between light absorption and charge recombination. These data‐driven insights guided the synthesis of representative materials, with experimental hydrogen evolution rates deviating by less than 5% from predictions. This work establishes a scalable and transferable paradigm, transforming fragmented literature into actionable intelligence and offering a powerful strategy for accelerating the development of functional materials.

## Graphical abstract

This work establishes a pipeline that transforms fragmented literature into a structured database for graphitic carbon nitride photocatalyst discovery. A prompt‐engineered, cross‐model large language model ensemble automates high‐fidelity extraction, enabling interpretable machine learning to identify dominant performance descriptors. These data‐driven insights directly guide synthesis, with experimental validation confirming predictions. The framework provides a scalable, domain‐general strategy for AI‐accelerated materials science.

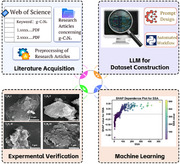

## 1 Introduction

For decades, the development of advanced materials has depended on empirical correlations and data‐driven modeling. The recent integration of machine learning (ML) has significantly enhanced our capacity for predictive modeling and inverse design, yet these approaches fundamentally require systematically organized data [1]—a requirement that remains largely unmet as critical materials information continues to be embedded within unstructured text across scientific literature. This persistent data fragmentation, intensified by the exponential expansion of scientific literature, presents a critical bottleneck for accelerating materials discovery [2, 3, 4]. While rule‐based tools, (e.g., ChemDataExtractor [5], OSCAR [6], ChemicalTagger [7]) partially address this challenge, their reliance on shallow models [8, 9, 10, 11, 12, 13, 14, 15], manual rules [16], and extensively preprocessing limits their robustness across complex, domain‐specific content [17], thereby substantially limiting the pace of materials innovation [18].

The emergence of large language models (LLMs) marks a transformative shift, offering a paradigm shift leveraging deep contextual understanding to automate high‐fidelity data extraction [19, 20, 21]. Unlike specialized ML approaches requiring extensive customization, LLMs provide a versatile foundation for converting textual information to structured knowledge. Pioneering systems like ChatExtract [22] and GPT‐4 [23]‐driven databases demonstrate this potential. However, their application often lacks the specialized tuning [24] needed for materials science's nuanced terminology and implicit data presentation, highlighting the crucial role of prompt engineering in achieving domain‐specific accuracy [25]. Systematic prompt engineering bridges this gap, serving as the key interface to guide LLM reasoning processes [26]. In materials science, this involves designing prompts that explicitly define technical terms, specify output formats, and interpret cross‐modal data. The CO‐STAR framework provides a structured approach [27] to ensure prompts are both interpretable and task‐oriented. Through iterative refinement [28], general‐purpose LLMs can be transformed into precise domain‐specific extraction tools.

To construct a comprehensive database for photocatalyst design, a principled approach for selecting the target synthesis parameters was implemented. The selection criteria were based on three pillars: i) domain significance—parameters must have an established theoretical or empirical link to photocatalytic performance, as supported by seminal reviews [29]; ii) reporting frequency—parameters must be consistently reported across a substantial fraction of the target literature to ensure dataset completeness [30]; and iii) extractability—parameters must be explicitly quantifiable or categorizable from text, tables, or figures [31]. Given the diverse strengths of contemporary LLMs, optimal performance requires strategic model selection based on architectural advantages [32], which reflects a broader trend toward leveraging specialized models or components. This is reflected in recent works that explore related strategies. For example, the X‐LoRA [33] framework combines low‐rank adapter experts to fine‐tune LLMs, dynamically mixing specialized adapters for specific domains. Similarly, SLERP‐based model [34] merging is used to combine multiple fine‐tuned models, unlocking new capabilities by blending their strengths. Additionally, the BioinspiredLLM [35] model focuses on bio‐inspired materials and demonstrates the potential of LLMs in creative design and hypothesis generation. These approaches illustrate the growing influence of strategies in enhancing LLM performance across specialized fields.

Multimodal capability proves particularly crucial for scientific extraction [36], as key materials parameters often reside in figures rather than text. While general‐purpose models (e.g., GPT‐4o [37], Claude 3.5) offer balanced performance with native multimodal support, specialized architectures excel in specific areas: long‐context specialists (e.g., Kimi [38]) process full research papers [39], and reasoning‐focused models (e.g., DeepSeek R1 [40]) enable structured output generation. This diversity motivates our model ensemble strategy, which integrates specialized models to dynamically route tasks, leveraging each model's strengths for enhanced performance [41]. Long‐context experts extract experimental narratives, multimodal models [42] decode graphical data, and reasoning specialists [43] synthesize insights into structured formats. Orchestrated through precision prompt engineering, this ensemble approach transcends single‐model limitations, significantly enhancing extraction accuracy and robustness.

The high‐quality structured database produced by this model ensemble strategy enables subsequent machine learning analysis [44] aimed at establishing reliable predictive relationships between material parameters and photocatalytic performance. The application of interpretable ML approaches, particularly SHapley Additive exPlanations (SHAP), facilitates the decoding of complex model behaviors to elucidate underlying physical chemistry principles, a strategy that has been successfully demonstrated in areas of battery materials design [45]. These data‐driven insights yield actionable design principles that can directly guide the optimization of synthesis protocols, as demonstrated by the robotic platform for the inverse design of colloidal nanocrystals [46] and the Bayesian optimization of polymer processing conditions guided by NMR data [47].

**SCHEME 1:**
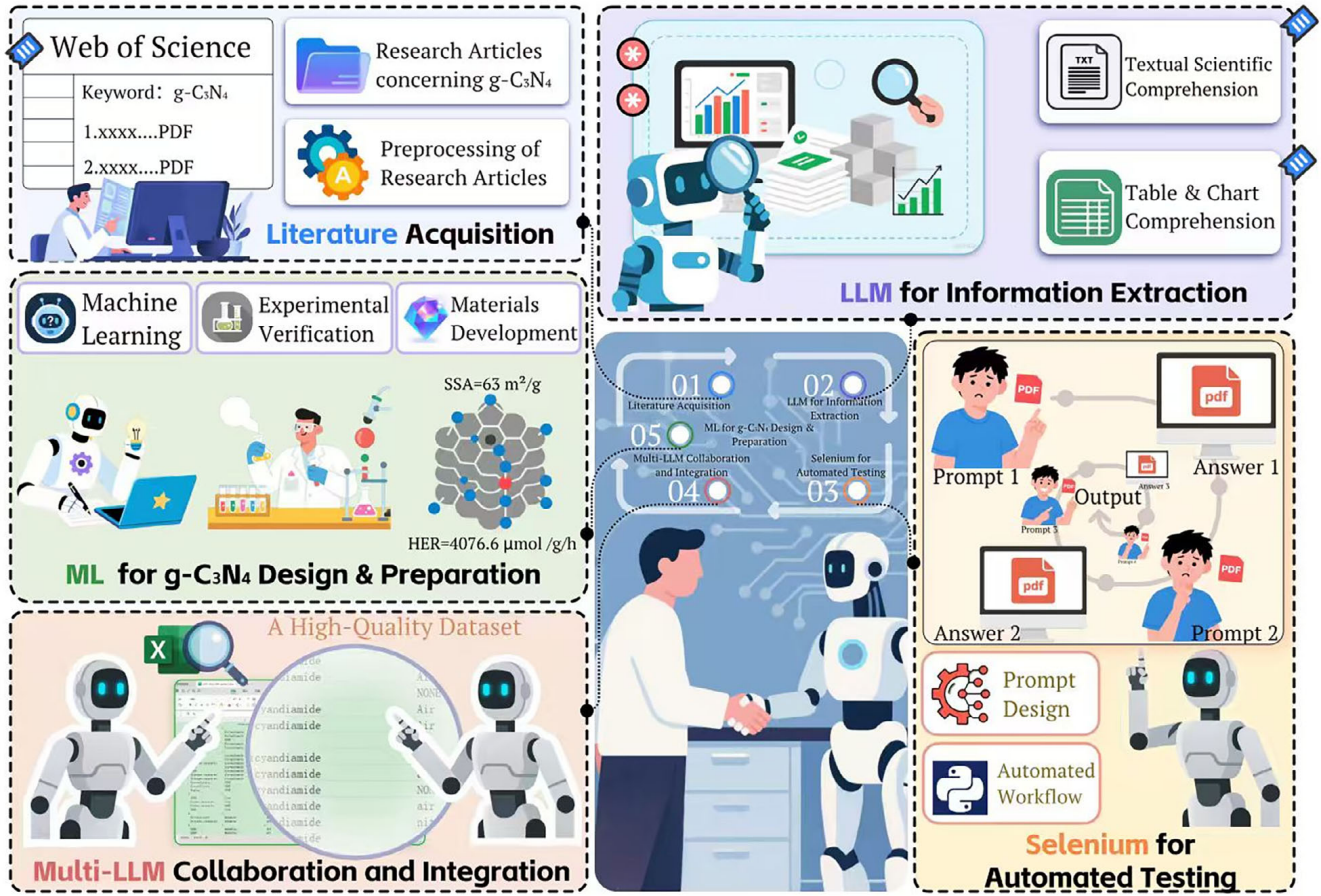
Schematic illustration of an automated g‐C_3_N_4_ literature mining and machine learning–integrated workflow based on multimodal LLM collaboration.

The methodology proves particularly valuable for graphitic carbon nitride (g‐C_3_N_4_) photocatalysts [48], where data fragmentation severely impedes progress. As a prominent visible‐light‐driven photocatalyst for hydrogen evolution [49, 50, 51, 52], g‐C_3_N_4_ suffers from charge recombination and limited light absorption. Although defect engineering through vacancies [53, 54, 55, 56, 57, 58, 59], doping [60, 61, 62, 63, 64, 65], or crystallinity control [66, 67, 68, 69, 70, 71] effectively enhances HER activity, critical synthesis‐performance relationships remain scattered across publications, preventing ML‐guided design. Herein, drawing on the principle of task‐specific model specialization, we propose a model ensemble strategy [72] to automate the construction of a high‐quality, structured database for defect‐engineered g‐C_3_N_4_ photocatalysts (Scheme 1). Our framework achieves 90% accuracy/recall in extracting synthesis parameters, and HER metrics. ML modeling of this dataset reveals quantitative nonlinear relationships between structural features (e.g., surface area, bandgap) and hydrogen evolution rates. Predictions were experimentally validated through targeted synthesis, confirming the model's reliability. This work establishes a scalable pathway for accelerating functional materials discovery and highlights the transformative role of LLMs in overcoming data fragmentation challenges across advanced materials research.

## 2 Results and Discussion

### 2.1 Overview of the Data Extraction Pipelines

Recent advances in text mining have enabled domain‐specific databases construction by extracting material synthesis methods and basic properties of materials from scientific literature. However, these efforts typically target limited parameters [73, 74, 75, 76], failing to address the multifaceted data landscape of defect‐engineered g‐C_3_N_4_. Here, synthesis conditions, defect types, and photocatalytic performance metrics are distributed across heterogeneous formats—including prose, tables, and figures‐introducing substantial complexity for systematic data aggregation. To overcome this, we propose multimodal vision ‐language models (VLMs), leveraging their integrated cross‐modal reasoning to concurrently parse textual and visual information. This approach enables holistic extraction of high‐dimensional material data previously inaccessible to conventional pipelines.

A collection of 175 research articles (Jan 2015‐Aug 2025) on defected‐engineered g‐C_3_N_4_ photocatalysts for hydrogen evolution were systematically compiled. The literature was sourced from Web of Science and six major publishers (Figure S1). To establish a comprehensive structure for data extraction, 19 critical parameters governing photocatalytic performance were identified. These encompass performance metrics (e.g., specific surface area, bandgap), synthesis parameters (precursor materials, heating temperature/rate), testing conditions (sacrificial agents, cocatalysts), and the photocatalytic hydrogen evolution efficiency. Detailed descriptions of all parameters are provided in Table S1.

**FIGURE 1:**
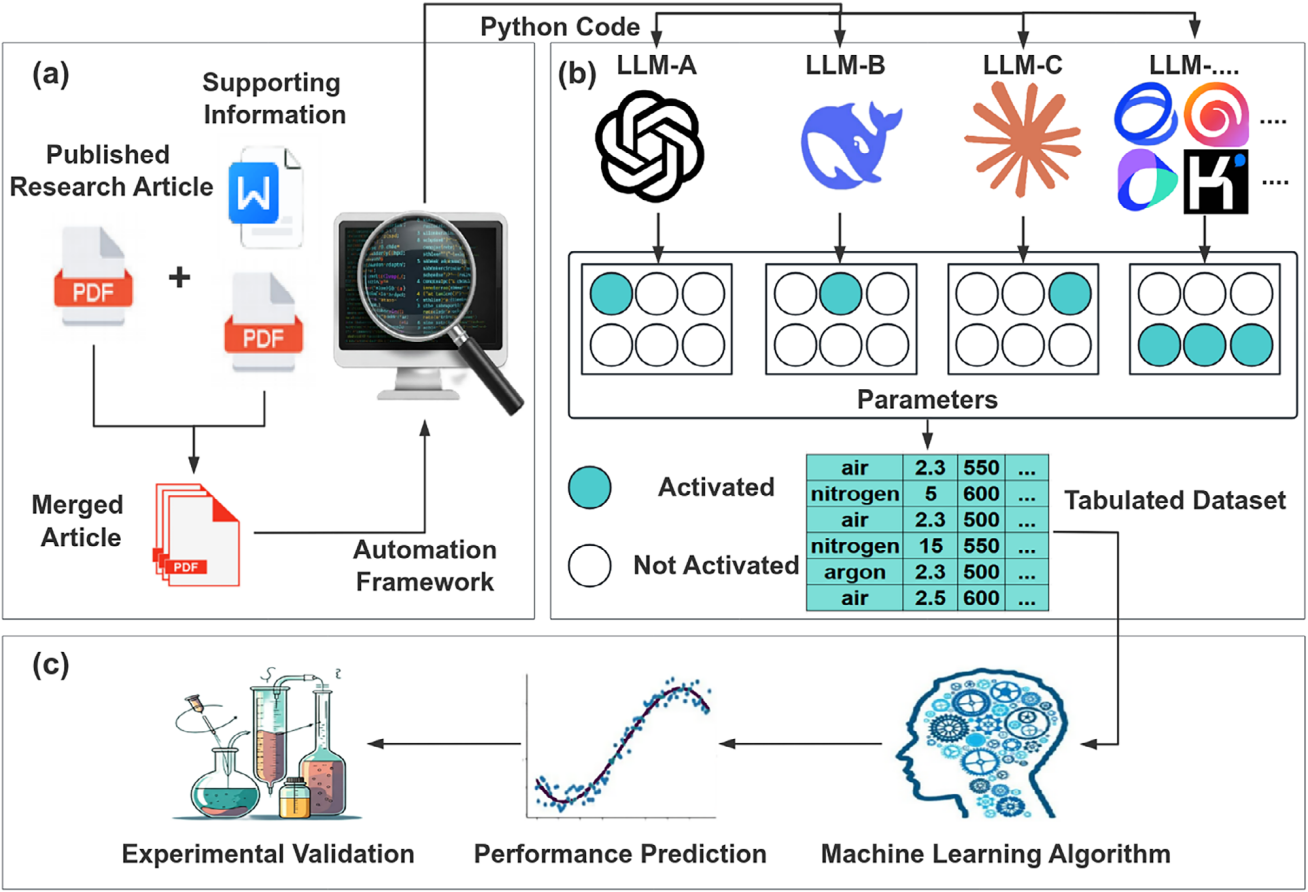
Overall workflow of data extraction. (a) Literature preprocessing and the construction of an automated data extraction pipeline. (b) Data extraction using multiple large language models with inter‐model collaboration for the integration of a high‐quality dataset. (c) Construction of machine learning models based on the obtained dataset, followed by experimental synthesis for model validation.

To address inconsistent data localization across publications—where performance parameters and testing conditions appear in main texts while synthesis methods often reside in the Supporting Information—an automated preprocessing pipeline were implemented (Figure S2). In this workflow, article PDFs and their Supporting Information files were programmatically merged into a single PDF document (Figure 1a). These consolidated files were then processed by a multi‐LLM extraction system, where Selenium‐based automation enabled batch processing at scale, eliminating manual intervention. A comparative analysis of the LLM outputs (Figure 1b) facilitated the construction of a high‐quality database for defective g‐C_3_N_4_ photocatalytic hydrogen production. This curated dataset subsequently provided the foundation for machine learning‐driven theoretical guidance, the accuracy of which was confirmed through experimental validation as summarized in Figure 1c.

### 2.2 Prompt Engineering and Workflow Automation

**FIGURE 2:**
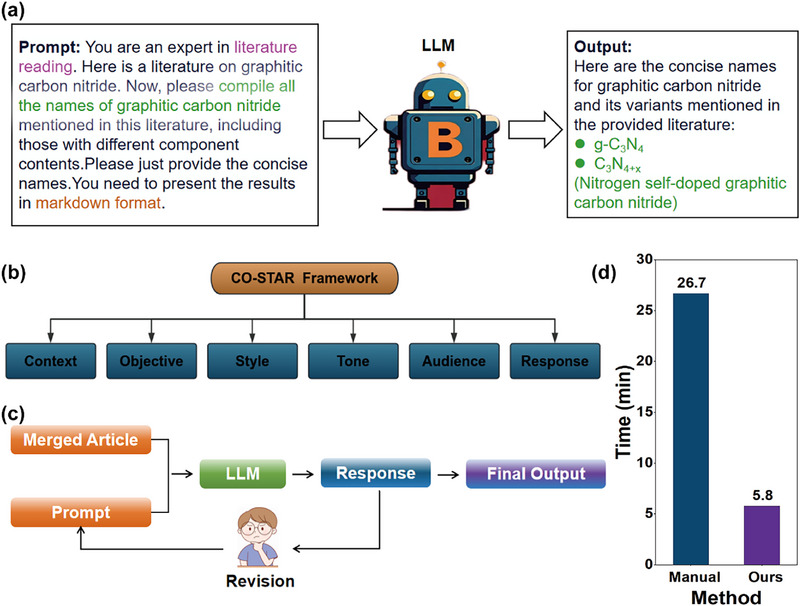
Overview of prompt design strategies and automation performance. (a) Example of prompt input and the corresponding output generated by the LLM, illustrating how the model responds to parameter‐specific instructions in the context of literature extraction. (b) Detailed description of the CO‐STAR‐based prompt design. (c) Schematic diagram showing how prompt design was iteratively refined based on manual observation of model outputs. (d) Comparison of the average execution time between our automated method and the manual approach.

The complex nature of materials science literature, with its specialized terminology and implicit data presentation, necessitates sophisticated information extraction approaches. A structured prompt engineering strategy was developed using the CO‐STAR framework, which systematically ensures clarity in technical definitions, task alignment, and structured output formatting through iterative refinement (Figure 2a; more details in Figure S3). This methodology produced optimized prompts for cross‐modal data extraction while maintaining interpretable logic throughout the automated workflow.

The implementation combined the CO‐STAR framework with XML delimiters to address data fragmentation challenges in materials informatics [77]. The framework's structured components—Context, Objective, Style, Tone, Audience, and Response format (Figure 2b)—ensured consistency across diverse scientific reporting styles, while XML tags enhanced parsing accuracy by clearly separating instructions from data inputs.

A zero‐shot prompting strategy was employed, wherein model responses iteratively refined subsequent queries. Through multiple refinement cycles (Figure 2c), prompts were systematically calibrated to address ambiguous phrasing that caused incomplete outputs. This process introduced explicit parameter definitions and structured guidance for numerical data recognition, substantially improving extraction accuracy while providing insights into LLM interpretation of scientific literature.

For scalable processing, an automated pipeline was developed to replace labor‐intensive manual processing. Benchmarking against expert manual extraction demonstrated a fivefold acceleration in processing time (Figure 2d), transforming data acquisition efficiency and reallocating researcher effort to strategic initiatives.

This methodology aligns with the evolution of LLMs in scientific workflows toward structured, agent‐based systems [78]. This trend, exemplified by specialized frameworks like ToPolyAgent and autonomous optimization approaches [79], conceptually aligns with the current model ensemble strategy and supports the development of closed‐loop, data‐driven materials discovery pipelines for defect‐engineered g‐C_3_N_4_ photocatalysts.

### 2.3 Performance Evaluation of Data Extraction and Model Collaboration

The foundation of a rigorous evaluation lies in a high‐quality benchmark. To this end, a ground‐truth dataset was established through the manual curation of 61 scientific publications, yielding 153 unique entries for defect‐engineered g‐C_3_N_4_, each annotated with up to 19 critical parameters. To ensure consistency, a systematic protocol for handling missing data was implemented: unreported atmospheric conditions were defaulted to air, and unquantified parameters were explicitly recorded as N/A. This meticulous approach to dataset construction echoes the emphasis on reliable ground truth in other data‐intensive scientific domains [80]. The automated extraction pipeline was then tasked with processing the identical publication set, with equivalent missing‐data logic embedded directly into the prompt design to ensure a fair comparison.

Performance was quantified using a ternary classification system—true positives, false positives, and false negatives—calculated for each parameter. This granular approach allowed for the computation of precision, recall, and F1 scores across all parameters, providing a multi‐faceted benchmark of system accuracy that moves beyond aggregate metrics to reveal parameter‐specific strengths and weaknesses.

**FIGURE 3:**
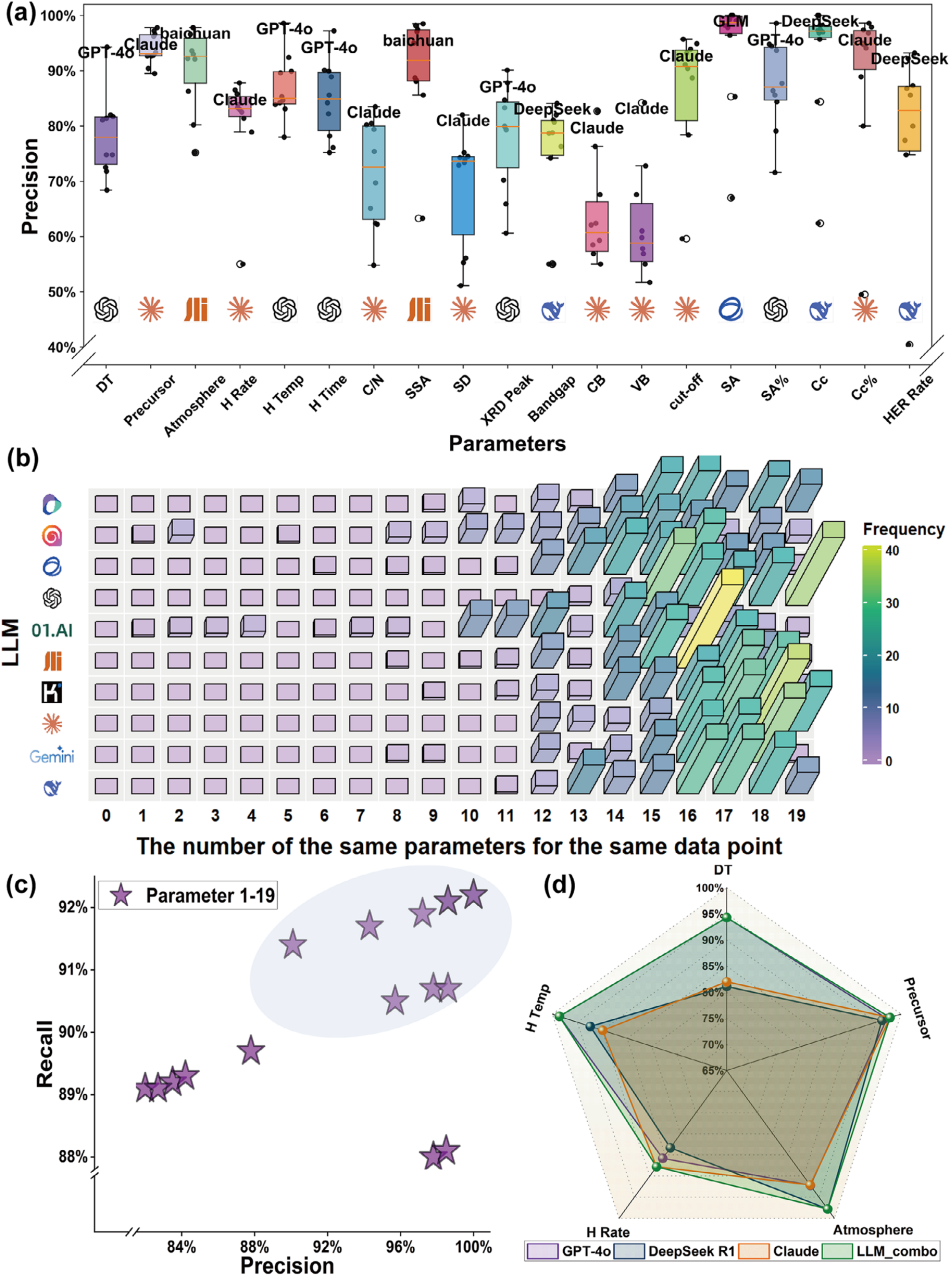
Performance evaluation of data extraction. (a) Box plots of precision for the extraction of different parameters by ten LLMs. (b) Frequency distribution heatmap used to assess the quality of individual data entries. (c) Precision and recall for each of the 19 parameters in the final integrated dataset. (d) Comparison of the LLM_Combo method with top‐performing individual models across multiple parameters.

Facing the inherent limitations of general‐purpose LLMs in a specialized domain, a model ensemble framework was implemented to strategically leverage model diversity. This decision is supported by recent research demonstrating that model ensemble architectures excel at integrating specialized capabilities for complex tasks [81]. Ten multimodal LLMs independently processed the benchmark set (see Methods for the list of models), and their performance heterogeneity was substantial (Figure 3a). A precision variance exceeding 30% was observed between the top and bottom models for key parameters. For instance, while GLM, GPT‐4o, and DeepSeek R1 achieved 100% precision in extracting the Sacrificial Agent, Claude significantly outperformed all peers on electronic band‐structure parameters (CB/VB). These quantified variations (Figures S4 and S5) were not merely recorded; they were instrumental in designing the dynamic expert routing logic of the model ensemble architecture, thereby transforming model heterogeneity from a challenge into a strategic advantage.

The assessment extended beyond standard metrics to evaluate per‐entry data quality via a frequency distribution heatmap (Figure 3b), which visualized the number of correctly extracted parameters per entry across all models. This analysis confirmed that all LLMs produced consistently high‐quality outputs, with detailed distributions available in Figure S6. Informed by these multidimensional insights, a final four‐LLM ensemble (LLM_Combo) was constructed, with parameter‐specific model assignments detailed in Table S2. The resulting model ensemble pipeline achieved exceptional accuracy: over 50% of parameters exceeded 90% precision/recall, and all remaining parameters surpassed 80% (Figure 3c). Crucially, the LLM_Combo ensemble demonstrated consistent outperformance across every parameter compared to any single model, a superiority quantitatively validated through radar chart analysis (Figure 3d).

The critical importance of multimodal understanding was unequivocally confirmed by a controlled comparison. When evaluated on the same 61 publications, the multimodal GPT‐4o consistently surpassed the text‐only GPT‐4 across all metrics (Figure S7). This performance gap underscores a fundamental aspect of materials science literature: key performance data, such as band energies and reaction kinetics, are often encoded within figures and diagrams rather than explicit text.

Our comparative analysis initially revealed an unexpected performance advantage of GPT‐4o over an earlier version of GPT‐5 for structured scientific data extraction (Figure S8). While counterintuitive given the presumed architectural advances of GPT‐5, this finding was consistent across repeated experiments. To further examine this observation, we evaluated a more recent model iteration (GPT‐5.2‐2025.12.11) under identical prompting conditions. The performance of this updated version was broadly comparable to that of GPT‐4o (Figure S9). We hypothesize that the initial discrepancy may be attributed to differences in model maturity and stability; the extensively refined GPT‐4o likely offers more predictable behavior for precise extraction, whereas earlier iterations of a new model series might exhibit instability or over‐interpretation that hinders structured output. Critically, this series of experiments underscores that incremental version upgrades of general‐purpose LLMs, without tailored optimization, do not automatically translate into significant gains for specialized tasks. This highlights the necessity of domain‐specific benchmarking over reliance on presumed architectural superiority when selecting tools for scientific AI workflows [82].

The final workflow synergistically integrated four strategically selected LLMs—GPT‐4o, Claude, Baichuan AI, and DeepSeek R1—each contributing distinct expertise. GPT‐4o delivered best‐in‐class cross‐modal reasoning. DeepSeek R1 exhibited exceptional interpretability, generating step‐by‐step rationales with explicit figure references and even surpassing GPT‐4o by 1% in accuracy on the key photocatalytic efficiency parameter. Claude matched GPT‐4o's performance across multiple metrics, while Baichuan AI provided robust domain‐specific extractions. This strategic integration, which aligns with the principle of routing tasks to specialized models, effectively transformed architectural diversity into collective performance gains, as conclusively shown in Figure 3d.

### 2.4 Machine Learning and Interpretability Analysis

To establish a predictive model for hydrogen evolution in defect‐engineered g‐C_3_N_4_, a machine learning approach was employed using the structured dataset [83]. The input features encompassed numerical and one‐hot encoded categorical variables, with photocatalytic hydrogen production efficiency as the target (see Table S3 and Figures S10 and S11 for feature details and data distributions). A logarithmic transformation was first applied to the target variable to mitigate its long‐tailed distribution, ensuring robust model training. Model performance was then evaluated using 10‐fold cross‐validation, a strategy designed to validate model generalizability.

**FIGURE 4:**
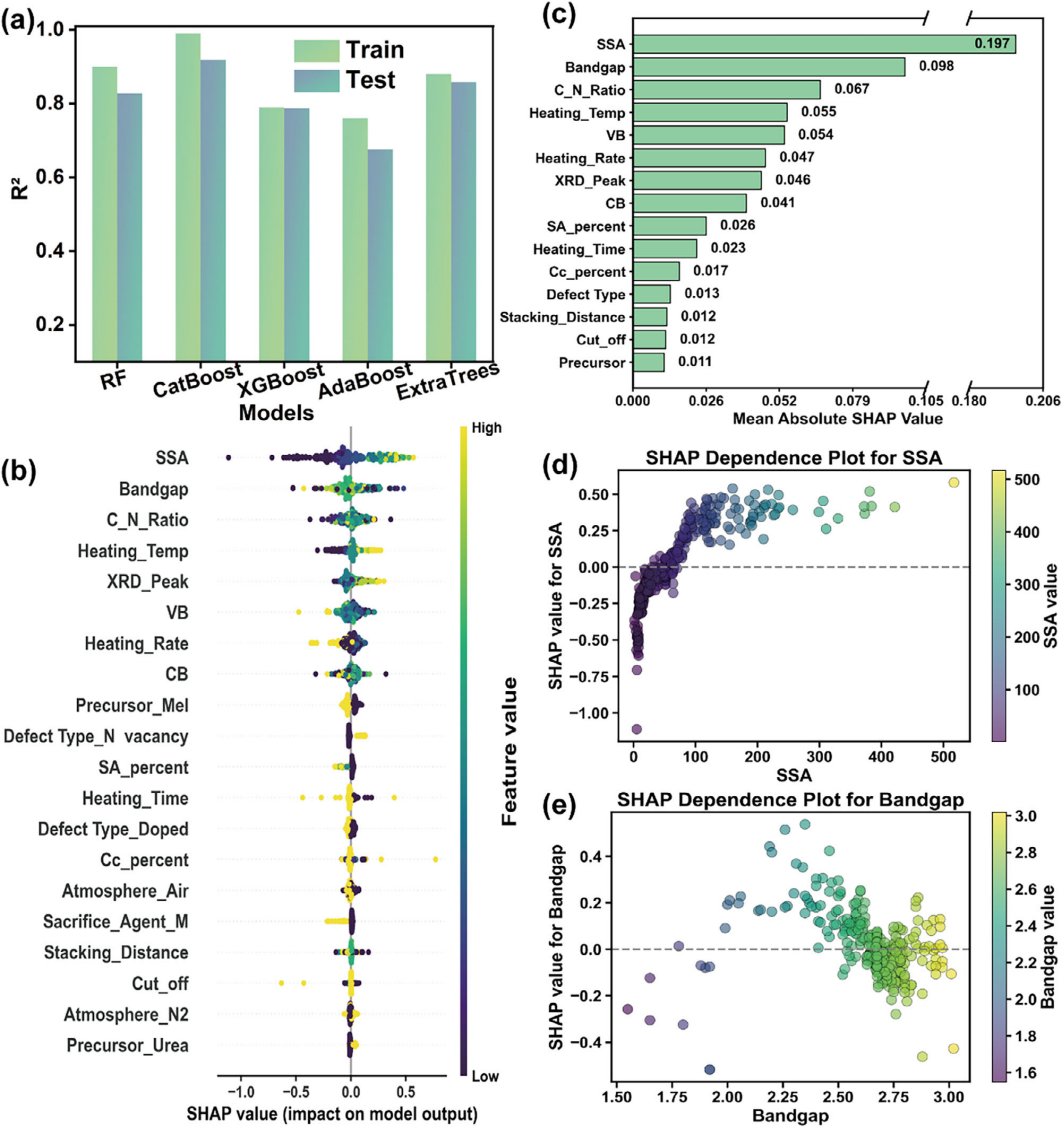
Performance of ML models and interpretability analysis using the SHAP method. (a) R^2^ of five ML models. (b) SHAP summary plot for feature importance. (c) Mean absolute SHAP values of grouped features. (d) SHAP dependence plot for SSA. (e) SHAP dependence plot for Bandgap.

The performance of five state‐of‐the‐art ensemble algorithms—XGBoost, Random Forest, CatBoost, AdaBoost, and ExtraTrees—was benchmarked (hyperparameter optimization in Figures S12–S18). Evaluation based on R^2^ and RMSE metrics identified CatBoost as the superior model, achieving exceptional performance on both training (RMSE: 0.075; R^2^: 0.99) and test sets (RMSE: 0.287; R^2^: 0.81) (Figure 4a, Table S4). Its strong generalization capability (test R^2^ = 0.81) underscores the high quality of the curated dataset and positions CatBoost as a reliable predictor for guiding photocatalyst design, a critical step forward from purely trial‐and‐error experimental approaches [84].

To transcend the “black box” nature of the model and extract physical insights, a SHAP analysis was conducted [85]. This interpretability analysis is pivotal, as it bridges data‐driven predictions with fundamental physical chemistry principles [86]. The SHAP analysis revealed that specific surface area (SSA) was the most dominant feature governing photocatalytic performance, followed by bandgap as a secondary thermodynamic factor (Figure 4b,c). This clear hierarchy immediately provides an actionable design principle: optimizing photocatalytic activity requires maximizing accessible surface sites while strategically tuning the electronic structure via bandgap engineering (Figures S19–S22).

Delving deeper, SHAP dependence analysis revealed a critical nonlinear relationship between SSA and photocatalytic hydrogen evolution efficiency (Figure 4d). The SHAP value transitioned from negative to positive as SSA increased, eventually plateauing. This trend reflects the underlying materials behavior: moderate SSA expansion enhances active site density and charge separation, boosting performance. However, beyond an optimal threshold (∼170 m^2^ g^−1^), diminishing returns occur due to active site saturation and the emergence of competing limitations like reduced light absorption. This insight, gleaned directly from the model, underscores the necessity of a balanced structural design rather than blindly pursuing maximal SSA [87].

A similarly instructive non‐monotonic relationship was observed for bandgap (Figure 4e). SHAP values were most favorable at narrower bandgaps (2.4 eV), became less positive, and eventually turned negative as the bandgap widened beyond ∼2.7 eV. This “increase‐then‐decrease” trajectory confirms a fundamental trade‐off in photocatalyst design: a narrow bandgap favors light absorption but risks rapid charge recombination, while a wide bandgap impedes light harvesting. The identified optimal range of approximately 2.2–2.4 eV provides a quantitative target for synthesis efforts.

Finally, the SHAP‐derived relationships were formalized into empirical functions linking HER to SSA and bandgap (Equations (1), (2)). These functions serve as a practical screening heuristic, establishing quantitative design targets of SSA 170 m^2^ g^−1^ and bandgap ≈2.31 eV for high‐performance g‐C_3_N_4_ (Figures S23 and S24). By translating complex model insights into simple, actionable thresholds, this approach significantly streamlines the initial screening process in photocatalyst development. The underlying physical chemistry principles uncovered by interpretable ML thus form a solid foundation for these actionable design principles [88], moving the field toward a more rational and accelerated discovery pipeline.

(1) $$HER_{pred}\left(SSA\right)\approx 44.58\times {\left(SSA+6.421\right)}^{0.626}$$

(2) $$\begin{array}{c} HER_{pred}\left(Bandgap\right)\approx 0.235\times 10^{2.986\times Bandgap}\times 10^{-0.647\times Bandgap^{2}} \end{array}$$

### 2.5 Experimental Validation and Performance Evaluation

The ultimate test of any predictive framework lies in its successful guidance of experimental synthesis. To validate the machine learning framework, six defect‐engineered g‐C_3_N_4_ catalysts (C_3_N_4_‐I to C_3_N_4_‐VI) were synthesized, strategically spanning a range of specific surface areas (SSA) and bandgaps to broadly cover the model‐predicted parameter space. This approach of targeted synthesis, guided by computational prediction, is emblematic of the modern, accelerated materials discovery paradigm, moving beyond traditional trial‐and‐error methods.

**FIGURE 5:**
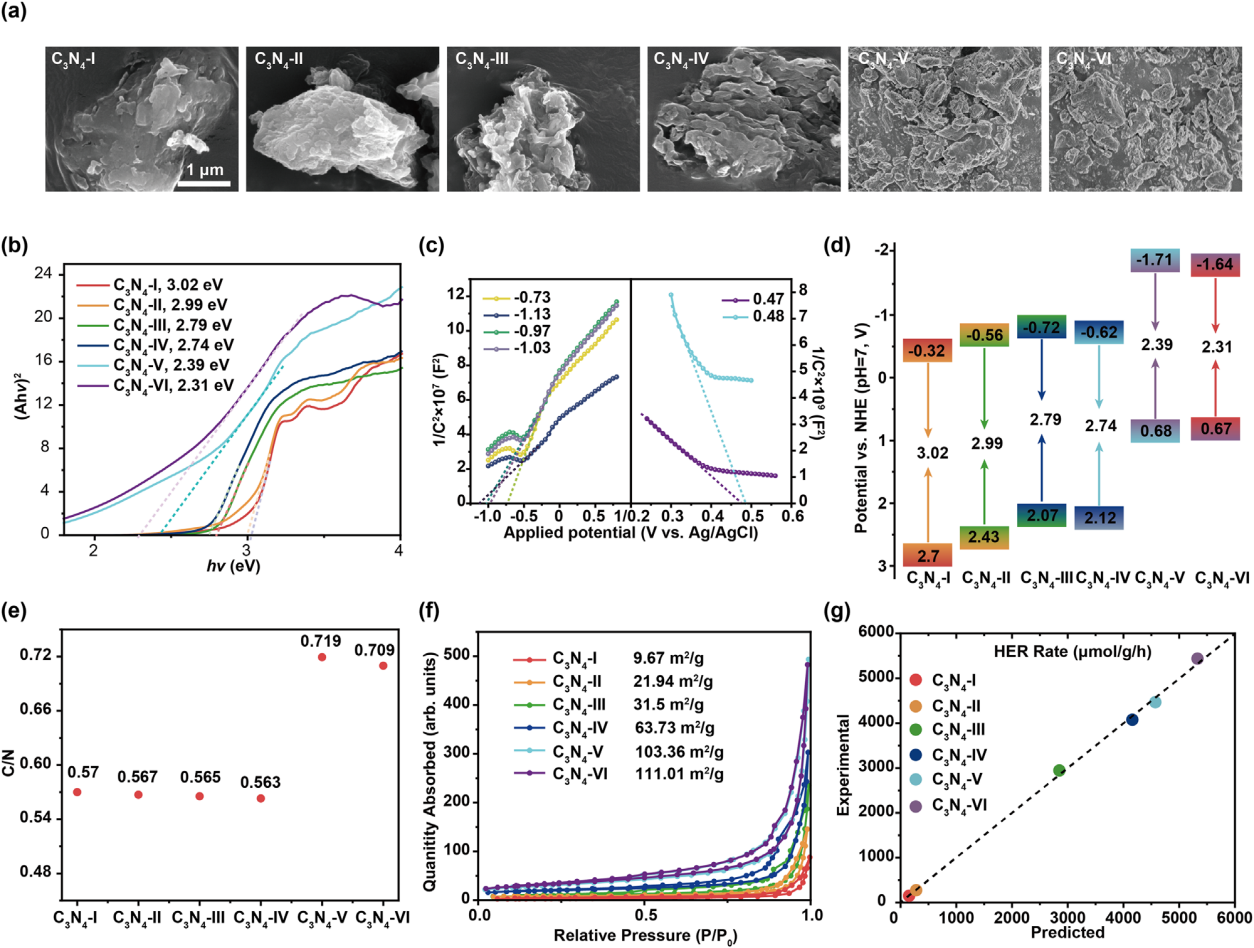
Characterization of morphology and physicochemical properties of g‐C_3_N_4_. (a) The SEM images, (b) The plots of (Ah*ν*)^2^ and h*ν*, (c) Mott‐Schottky curves, (d) Schematic illustration of band structure, (e) The C/N mass ratio characterized by elemental analysis, (f) N_2_ physisorption isotherms, and (g) HER rates (Predicted vs. Experimental) of different g‐C_3_N_4_.

A comprehensive suite of structural and optical characterizations confirmed that defect engineering had successfully introduced systematic variations across the catalyst series. X‐ray diffraction (XRD, Figure S25) confirmed the preservation of the characteristic graphitic carbon nitride phase in all samples, ensuring that performance differences stemmed from structural modifications rather than phase changes. Scanning electron microscopy (SEM, Figure 5a) revealed an increasingly wrinkled and porous morphology from C_3_N_4_‐I to C_3_N_4_‐VI, providing a visual correlate to the measured increase in SSA. Critically, the combination of Tauc plots derived from UV‐Vis spectra (Figure 5b; Figure S26) and Mott‐Schottky measurements (Figure 5c) allowed for the precise determination of the band structure for each catalyst. The resulting data (Figure 5d) pinpointed C_3_N_4_‐VI as possessing the narrowest bandgap (2.31 eV) and, along with elemental analysis (Figure 5e) and N_2_ adsorption‐desorption isotherms (Figure 5f), which confirmed its highest SSA, directly highlighted the two key parameters—bandgap and SSA—as being systematically tuned through our synthesis protocol, just as the model had identified as crucial.

The true validation of the data‐model pipeline emerged from photocatalytic hydrogen evolution testing (Figure 5g; Figure S27). The results showed a monotonic increase in activity from C_3_N_4_‐I to C_3_N_4_‐VI, a trend directly attributable to the synergistic effects of a progressively narrowing bandgap (enhancing light absorption) and an enlarging surface area (providing more active sites). This experimentally observed structure‐property relationship was fully consistent with the predictions made by the machine learning model. Furthermore, the agreement between the predicted and experimental hydrogen evolution rates was exceptional, with all samples showing relative deviations below 5%—well within the intrinsic uncertainty of photocatalytic testing. This strong predictive‐experimental concordance provides robust, empirical proof‐of‐concept for the entire workflow, from data extraction to model prediction.

## 3 Conclusions

The rapid discovery of functional materials is often hindered by the difficulty of extracting structured knowledge from unstructured scientific literature, particularly in materials science where critical synthesis and performance parameters are frequently embedded in free‐text descriptions. In this work, we developed a closed‐loop data‐model‐experiment framework to address this data fragmentation challenge and enable efficient derivation of material design principles from fragmented scientific literature.

Using automated literature mining, we constructed a standardized defect‐performance database for defect‐engineered g‐C_3_N_4_ photocatalysts. Machine learning analysis of the curated dataset identified specific surface area and bandgap as the dominant descriptors governing photocatalytic hydrogen evolution activity, with optimal values of ∼170 m^2^ g^−1^ and 2.2–2.4 eV, respectively. Further interpretation revealed a non‐monotonic influence of bandgap, reflecting the trade‐off between light absorption and charge recombination.

These data‐driven insights were experimentally validated through the synthesis and characterization of representative g‐C_3_N_4_ photocatalysts, which exhibited excellent agreement with model predictions. The close correspondence between predicted and measured hydrogen evolution rates confirms the reliability of the proposed data–model–experiment workflow, highlighting its potential as a generalizable framework for data‐driven materials discovery.

In summary, this work delivers more than an isolated case study; it establishes a scalable and transferable template for accelerating functional materials discovery. By seamlessly integrating automated knowledge extraction with interpretable machine learning and experimental validation, our approach converts fragmented scientific literature into actionable design intelligence. This pipeline thus presents a powerful strategy to expedite the development of advanced materials, from energy storage to heterogeneous catalysis, offering a concrete realization of AI‐accelerated materials science.

However, certain limitations guide the current design of our framework. It relies heavily on prompt engineering and model ensemble strategy, rather than employing deeper architectural modifications such as fine‐tuning or implementing specialized routing frameworks. While our approach has proven effective, leveraging true task‐specific routing architectures could potentially offer further improvements in performance and efficiency. Additionally, with the increase in data volume and the improvement in data quality, fine‐tuning individual models or integrating agent‐based workflows, such as those used in autonomous scientific discovery, could help optimize our framework and enhance its adaptability across different materials domains. These methods would enable more precise and efficient data extraction, offering additional layers of flexibility and accuracy. In future, it is planned to explore these advanced techniques, including model fine‐tuning, more specialized routing architectures, and agentic AI, to further enhance the performance and scalability of our framework.

## 4 Methods

### 4.1 Data Collection and Processing

To investigate factors influencing photocatalytic hydrogen production in defective g‐C_3_N_4_ and enable machine learning‐guided experimental design, relevant parameters were systematically extracted from published literature to construct a comprehensive database. Publications related to g‐C_3_N_4_ were retrieved from Web of Science and subsequently downloaded. To ensure research timeliness, publications were limited primarily to the last decade (2015–2025). Given that performance parameters and hydrogen evolution efficiency typically appear in main texts while synthesis details frequently reside in Supporting Information, both components were incorporated for analysis. A total of 175 research articles were processed, with main texts and supplementary materials programmatically merged into unified documents (Figure S2). Vision‐language models were subsequently employed for cross‐modal data extraction from integrated textual content, tables, and figures.

### 4.2 Evaluation of Data Extraction

To evaluate the effectiveness of the proposed method in extracting parameters from literature on defective g‐C_3_N_4_‐based photocatalytic hydrogen production, precision, recall, and F1‐score are primarily employed as performance metrics, defined as follows:

(3) $$Precision=\frac{TP}{TP+FP}$$

(4) $$Recall=\frac{TP}{TP+FN}$$

(5) $$F1=\frac{2\times Precision\times Recall}{Precision+Recall}$$

In the task of text mining for parameters related to defective g‐C_3_N_4_ photocatalytic hydrogen production, each extracted parameter is categorized into one of three labels: TP (accurately extracted parameter), FP (incorrectly extracted parameter or irrelevant information extracted), or FN (parameter not successfully extracted). Precision quantifies the accuracy of parameter extraction, while recall evaluates the completeness of the extracted parameters. The F1‐score, derived from both precision and recall, provides a balanced measure of the overall performance of the extraction method.

### 4.3 Prompt Design

To ensure experimental consistency and enable fair comparison of model capabilities, all ten LLMs were provided with identical prompts. Although certain models (e.g., GPT‐4o) exhibited more accurate interpretation of the same instructions, this uniform input strategy allowed us to attribute performance differences to model architecture and capability rather than to prompt variation.

### 4.4 Automated Interaction Pipeline

The initial manual interaction protocol required sequential human operations: document uploading to the model interface, iterative prompt input for parameter extraction, response parsing, and Excel spreadsheet population—repeated per article. To overcome this bottleneck, an automated pipeline was developed using Python integrated with the Selenium browser automation framework. This system programmatically simulates the full interaction cycle, including document uploads, prompt transmission, response capture, and structured Excel output generation.

Batch processing of preprocessed articles was implemented, wherein parameter‐specific prompts are autonomously dispatched to the LLM, responses parsed, and results stored in structured tables. Upon article completion, the workflow automatically advances without intervention. This approach significantly enhances processing efficiency while ensuring strong generalizability across LLM interfaces, enabling scalable, reproducible literature data extraction with minimal reconfiguration.

### 4.5 LLMs and Access Strategy

LLMs with visual understanding capabilities were employed for multimodal information extraction (GPT, Doubao, Hailuo, ChatGLM, 01AI, Baichuan, Kimi, Claude, Gemini, DeepSeek), including both proprietary commercial models (e.g., GPT‐4o from OpenAI; Claude 3 from Anthropic) and open‐source implementations (e.g., DeepSeek R1, ChatGLM). A complete list of all models tested, along with their specific versions and access dates, is provided in Table S5. Crucially, all models were exclusively accessed through their official web‐based interfaces without API integration, fine‐tuning, or local deployment. This uniform interface ensured consistent interaction protocols across diverse models and eliminated the infrastructure overhead associated with custom deployments. In addition, it facilitated direct, side‐by‐side visual comparison between model outputs and human expert annotations within the same environment, supporting a consistent evaluation of multimodal reasoning and extraction accuracy.

### 4.6 Cross‐Model Integration of LLMs

To leverage the distinct strengths of various LLMs, we implemented a performance‐driven model ensemble strategy for data extraction. Instead of using a single model for all parameters, we assigned each specific parameter type to the LLM that demonstrated the highest accuracy for that parameter during our validation phase. This specialization ensured that every data point was extracted by the most capable model for that particular task. Specifically, GPT‐4o was used to extract parameters 1, 5, 6, 10, 15, 16, and 17; Claude was used for parameters 2, 4, 7, 9, 12, 13, 14, and 18; BaichuanAI was used for parameters 3 and 8; and DeepSeek R1 was used for parameters 11 and 19. Each of the four models was applied to all relevant literature for automated data extraction, after which only the parameter types assigned to each model were retained. The selected parameters from different models were then merged to construct the final unified dataset. This procedure was designed to elevate the overall data quality for subsequent applications, such as downstream machine learning and domain‐specific dataset development.

### 4.7 Statistical Analysis

A structured pre‐processing pipeline was implemented prior to machine learning modeling. Numerical features were categorized into two groups: key numerical features (central to the model's predictive goal) and general numerical features. Missing values were handled accordingly: for general numerical features, they were imputed with the median of the corresponding feature; for key numerical features, a k‐nearest neighbors (KNN) interpolation (k = 5, using Euclidean distance) was applied. Categorical features with missing entries were assigned a dedicated “missing” category before undergoing one‐hot encoding. The target variable (e.g., hydrogen evolution rate) was log10‐transformed to approximate a normal distribution and stabilize variance. All pre‐processing was performed using Python. The complete code, along with a requirements.txt file specifying all dependencies and environment details, is available in the public repository linked in the Data Availability Statement.

### 4.8 Evaluation of ML Models

The performance of the machine learning models was evaluated using two commonly used metrics: R^2^ and RMSE. The R^2^ score reflects the proportion of the variance in the dependent variable that is predictable from the independent variables, and is calculated as:

(6) $$R^{2}=1-\frac{\sum_{i=1}^{n}{\left(y_{i}-\hat{y_{i}}\right)}^{2}}{\sum_{i=1}^{n}{\left(y_{i}-\bar{y}\right)}^{2}}$$

where *y_i_* is the true value, $\hat{y_{i}}$ is the predicted value, and $\bar{y}$ is the mean of the true values.

The RMSE measures the average magnitude of prediction errors and is computed as:

(7) $$\mathrm{RMSE}\;=\sqrt{\frac{1}{n}\sum_{i=1}^{n}{\left(y_{i}-\hat{y_{i}}\right)}^{2}}\;$$

A higher R^2^ value (closer to 1) and a lower RMSE indicate better model performance.

## Conflicts of Interest

The authors declare no conflicts of interest.

## Supporting information




**Supporting File**: advs74454‐sup‐0001‐SuppMat.docx.

**Supporting File**: advs74454‐sup‐0002‐Data.zip.

## Data Availability

The data that support the findings of this study are available from the corresponding author upon reasonable request.
