# Supplementary material for: Prompt Engineering Accelerates the Data‐Driven Discovery of Photocatalysts via an LLM‐Based Model Ensemble Strategy
Source: Adv Sci (Weinh). 2026 Feb 16;13(24):e24215. doi: 10.1002/advs.202524215 (PMC13116308; doi:10.1002/advs.202524215)
Supplement: Supplementary file 1 — Supporting File: advs74454‐sup‐0001‐SuppMat.docx. [file ADVS-13-e24215-s001.docx]

Supporting Information

**Prompt Engineering Accelerates the Data-Driven Discovery of Photocatalysts via an LLM-Based Model Ensemble Strategy**

*Dianyuan Li^a,b⊥^, Xichen Sun^a,b⊥^, Shaohua Sun^a,b⊥^, Runzhou Wang^c^, Miaomiao Zhang^a,b^, Meng Xiao^a,b^, Yue Wang^a,b^, Yuezhou Zhang^a,b^**

Supplemental Notes

Supplemental Notes 1

To enhance the consistency and validity of the constructed database, while minimizing the complexity of prompt design during large language model-based data extraction, we initiated the process with a selective screening of relevant papers. Given that papers on non-defective g-C_3_N_4_ can cause significant interference with those on defective g-C_3_N_4_ during the retrieval process. Therefore, to ensure both the quality and breadth of the selected papers, we decided to start with a review article on defective g-C_3_N_4_ and focused on the various types of defects summarized in the tables presented within the review. Building upon this framework, we progressively searched for and downloaded relevant research articles from the Web of Science database. However, in these papers, defective g-C_3_N_4_ was applied in various contexts, including the hydrogen evolution reaction (HER), nitrogen reduction reaction (NRR), and oxygen evolution reaction (OER), among others. As a result, we conducted a round of manual screening to obtain related research articles containing the concerned information.

We obtained 175 papers and their supplementary files from over 20 different journals across six publishing groups: the American Chemical Society (ACS), Elsevier, the Royal Society of Chemistry (RSC), IOP Publishing, Springer, and Wiley. These papers were published between 2015 and 2025 and downloaded in PDF format. They collectively represent a broad spectrum of defective g-C_3_N_4_ materials and various writing styles.

Supplemental Notes 2

One-hot encoding is a commonly used method for converting categorical variables into binary vectors, where each category is represented by a unique position set to 1, with all other positions set to 0. In this study, one-hot encoding was applied to transform categorical features for the construction of machine learning models.

Hyperparameter Optimization: In this study, we utilized the Optuna framework to perform hyperparameter optimization for machine learning models. Optuna is an efficient and flexible optimization tool that uses a sampling-based approach to explore the hyperparameter space, enabling automated and scalable tuning. Its ability to prune unpromising trials early and allocate resources adaptively reduces the need for manual intervention.

Random Forest is an ensemble learning method that constructs multiple decision trees and aggregates their results to improve predictive performance and reduce overfitting. Each tree is trained on a bootstrap sample drawn from the original dataset, introducing variability and promoting robustness.

XGBoost is a gradient boosting framework that builds an ensemble of decision trees in a sequential manner, where each new tree aims to correct the errors made by the previous ones. It incorporates regularization techniques to control model complexity and prevent overfitting, and is known for its high computational efficiency and scalability. During training, XGBoost minimizes a regularized loss function, allowing for both accuracy and generalization.

CatBoost is a gradient boosting algorithm that is particularly well-suited for handling categorical features. It builds decision trees sequentially and incorporates techniques such as ordered boosting and categorical feature encoding to reduce overfitting and improve accuracy. CatBoost requires minimal preprocessing of categorical variables and is known for its strong performance with relatively little parameter tuning.

AdaBoost is an ensemble learning algorithm that combines multiple weak learners, typically shallow decision trees, in a sequential manner. Each learner is trained to focus on the errors made by the previous ones by assigning higher weights to misclassified samples. The final prediction is made through a weighted combination of all learners, allowing the model to gradually improve performance and reduce bias.

ExtraTrees is an ensemble learning method that builds multiple decision trees using the full dataset and introduces additional randomness in the node-splitting process. Unlike traditional decision trees or random forests, ExtraTrees selects split thresholds at random rather than searching for the most optimal split, which increases diversity among trees and often leads to improved generalization. It is known for its high training speed and robustness to overfitting.

Supplemental Notes 3

Synthesis of g-C_3_N_4_

3 g precursors (melamine, dicyandiamide, urea) were put into porcelain cup with a cap, then calcined at different temperature (500-600 °C) for different hours (3-4 h) with different heating rate (2-5 °C min^-1^) in a muffle furnace in air. After heating, the resulting yellow agglomerates were gently ground and treated under ultrasonication for 3 h as an aqueous solution (1 g L^-1^). Then the powder was filtered through a 0.45 μm membrane, washed three times by deionized water, and dried at 80 °C for further tests.

Characterization

X-ray diffraction (XRD) patterns were recorded on a D8 Advance diffractometer (Bruker, Germany, Cu Kα, λ = 1.54056Å) operated at 40 kV and 200mA at room temperature. The surface morphology and element mapping of CN were using a field emission scanning electron microscope (FE-SEM, ZEISS Gemini SEM 300) after lyophilization. The diffuse reflectance UV-vis (DR UV-vis) spectra were recorded on the UV-vis spectrophotometer (UV-3600 Plus, Shimadzu). Elemental analysis of g-C_3_N_4_ was conducted on a UNICUBE analyzer (Elementar, Germany). N_2_ adsorption isotherms experiments were carried out on the Micromeritics, TriStar II 3020 analyser at 77 K. Specific surface area was calculated by the Brunauer-Emmett-Teller (BET) method.

H_2_ generation ability of g-C_3_N_4_

20 mg g-C_3_N_4_ was dispersed in 20 mL of 3 wt% H_2_PtCl_6_ in methanol (20 vol%) and irradiated with Xe lamp cut-off filter 420 (λ > 420 nm,0.5 W cm^-2^,1 h) in N_2_ atmosphere. The amount of H_2_ produced is analyzed by gas chromatography after the reaction.

Supplemental Tables

**Table S1.** Description of different parameters extracted by LLMs.

| Index | Parameter | Description |
| --- | --- | --- |
| 1 | Defect Type | Atomic- or electronic-scale imperfections in the crystal structure of C_3_N_4_. |
| 2 | Precursor | The initial raw materials for synthesizing C_3_N_4_ can be converted into C_3_N_4_ with varied crystal structures, morphologies, and defect states through pyrolysis or calcination methods. |
| 3 | Atmosphere | The environmental atmosphere used for high-temperature calcination to prepare C_3_N_4_, such as N_2_, air, etc. |
| 4 | Heating Rate | The temperature increases per unit time (typically °C/min) during thermal treatment processes in photocatalyst synthesis. |
| 5 | Heating Temperature | The maximum processing temperature during thermal treatment in photocatalyst synthesis. |
| 6 | Heating Time | The time interval during which the photocatalyst precursor is maintained at the target temperature in thermal treatment processes. |
| 7 | C : N（Atomic Ratio） | C/N atomic ratio quantifies the stoichiometric relationship between carbon and nitrogen atoms in carbon nitride photocatalysts. |
| 8 | Specific Surface Area | Specific surface area is defined as the total accessible surface area per unit mass of a photocatalyst. |
| 9 | Stacking Distance | Stacking distance refers to the interplanar spacing between adjacent atomic layers in layered photocatalysts. |
| 10 | XRD Peak | XRD peaks are the characteristic diffraction signals generated when X-rays interact with the crystallographic planes of photocatalysts. |
| 11 | Bandgap | Bandgap is the minimum energy required to excite an electron from the valence band to the conduction band in semiconductor photocatalysts. |
| 12 | CB | Conduction Band (CB) is the lowest range of vacant electronic states above the Fermi level in semiconductor photocatalysts. |
| 13 | VB | Valence Band (VB) represents the highest range of occupied electronic states below the Fermi level in semiconductor photocatalysts. |
| 14 | cut-off | The cut-on wavelength of light sources employed in photocatalysis. |
| 15 | Sacrifice Agent | Sacrificial Agent refers to a chemical substance that is preferentially oxidized or reduced in a reaction, consuming photogenerated charge carriers (electrons or holes) to protect the target reaction or enhance the efficiency of the photocatalytic system. |
| 16 | Sacrifice Agent Concentration | The molar or mass ratio of the sacrificial agent in a unit reaction system. |
| 17 | Cocatalyst | A substance that does not directly participate in light absorption but significantly enhances the efficiency of photocatalytic reactions. |
| 18 | Cocatalyst Concentration | The molar or mass ratio of the Co-catalysts in a unit reaction system. |
| 19 | HER Rate | HER Rate is the key quantitative metric for evaluating photocatalytic hydrogen evolution reaction (HER) performance, representing the amount of hydrogen gas produced (μ mol) per unit mass of catalyst (g) per hour (h). |

**Table S2.** Model assignment for specific parameters in LLM_Combo.

| LLM | Parameter |
| --- | --- |
| GPT-4o | 1.5.6.10.15-17 |
| Claude | 2.4.7.9.12-14.18 |
| baichuanAI | 3.8 |
| DeepSeek R1 | 11.19 |

**Table S3.** Summary of Value Ranges for All Parameters Used in the Model.

| Index | Parameter | Parameter Space |
| --- | --- | --- |
| 1 | Defect Type | N vacancy, C vacancy, Doped, Crystallinity, Functional group |
| 2 | Precursor | DCDA, Mel, Urea, CA |
| 3 | Atmosphere | Air, N_2_, Ar |
| 4 | Heating Rate | [1, 50] (°C min^-1^) |
| 5 | Heating Temperature | [300, 650] (°C) |
| 6 | Heating Time | [1.5, 6] (h) |
| 7 | C：N（Atomic Ratio） | [0.512, 0.877] |
| 8 | Specific Surface Area | [1.35, 517] (m^2^ g^-1^) |
| 9 | Stacking Distance | [0.035, 0.336] (nm) |
| 10 | XRD Peak | [26.3, 29.8] (°) |
| 11 | Bandgap | [1.55, 3.02] (ev) |
| 12 | CB | [-1.89, -0.21] (ev) |
| 13 | VB | [0.49, 2.44] (ev) |
| 14 | cut-off | [300, 440] |
| 15 | Sacrifice Agent | TEOA, MeOH |
| 16 | Sacrifice Agent Concentration | Percentage of TEOA [10, 25] (%) |
| 17 | Cocatalyst | Pt, Pd, Au |
| 18 | Cocatalyst Concentration | Percentage of Pt [0.1, 8.7] (%) |
| 19 | HER Rate | [1.6, 22650] (μ mol g^-1^ h^-1^) |

**Table S4.** R^2^ and RMSE of training and test sets for five machine learning models.

| model | Training dataset | | Test dataset | |
| --- | --- | --- | --- | --- |
|  | R^2^ | RMSE | R^2^ | RMSE |
| RF | 0.90 | 0.207 | 0.72 | 0.349 |
| CatBoost | 0.99 | 0.075 | 0.81 | 0.287 |
| XGBoost | 0.79 | 0.300 | 0.68 | 0.374 |
| AdaBoost | 0.76 | 0.325 | 0.57 | 0.434 |
| ExtraTrees | 0.88 | 0.230 | 0.75 | 0.332 |

**Table S5.** Large language models used in this study and their version identifiers.

| LLM | version identifier |
| --- | --- |
| GPT-4o | GPT-4o-2024.11.20 |
| Doubao | Doubao-2024.05.15 |
| Hailuo | abab-6.5-2024.04.17 |
| GLM | GLM-4-2024.05.20 |
| 01AI | Yi-Large-2024.05.13 |
| Baichuan | Baichuan3-Turbo-2024.05.22 |
| Kimi | Kimi-2024.07.10 |
| Claude | Claude Sonnet 3.5-2024.11.13 |
| Gemini | Gemini 1.5 Pro-2024.10.31 |
| DeepSeek | DeepSeek R1-2025.01.20 |
| GPT-4 | GPT-4-2024.05.04 |
| GPT-5 | GPT-5-2025.08.07 |
| GPT-5 | GPT-5.2-2025.12.11 |

Supplemental Figures


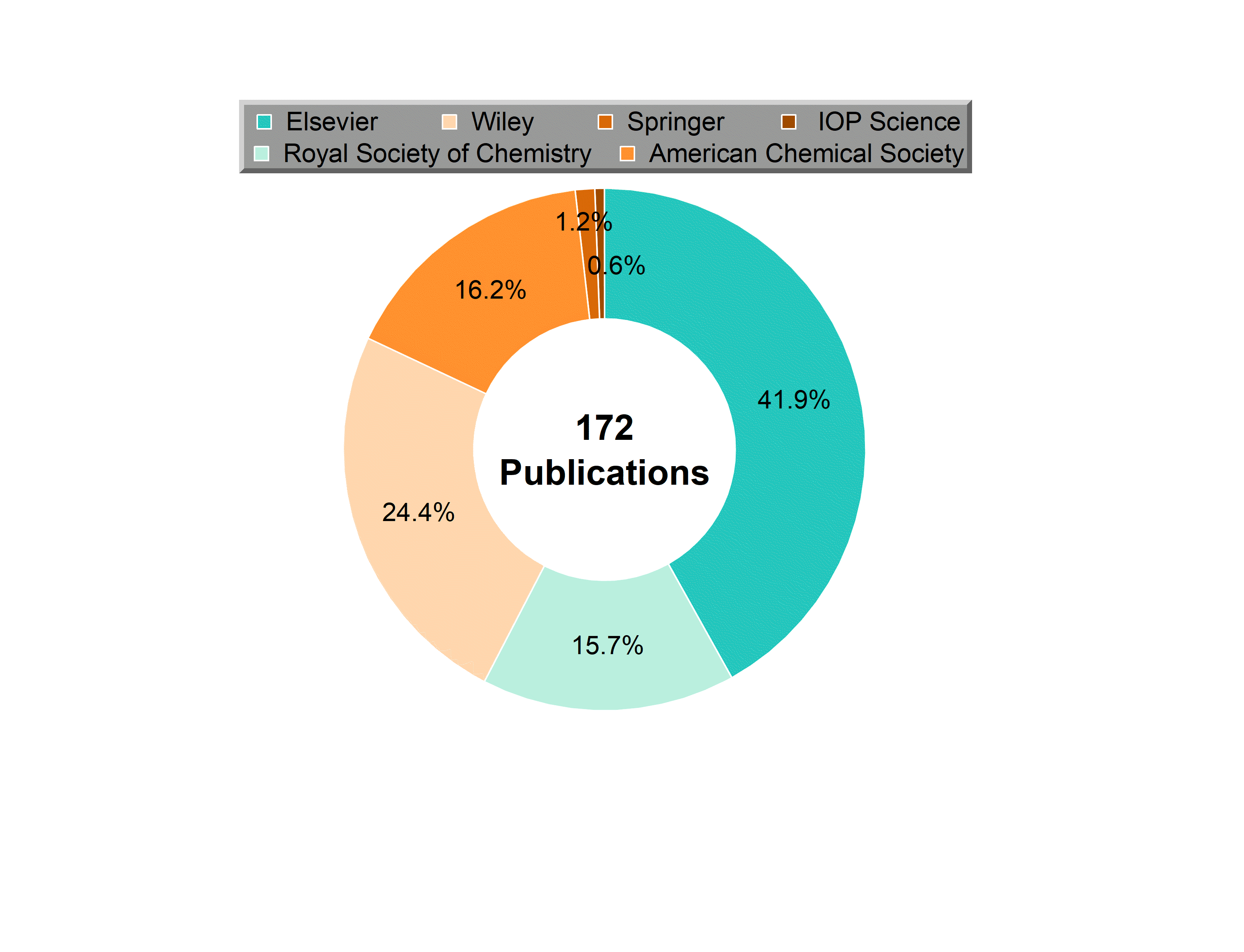


**Figure S1.** Distribution of the publishers in the academic literature used in our research.


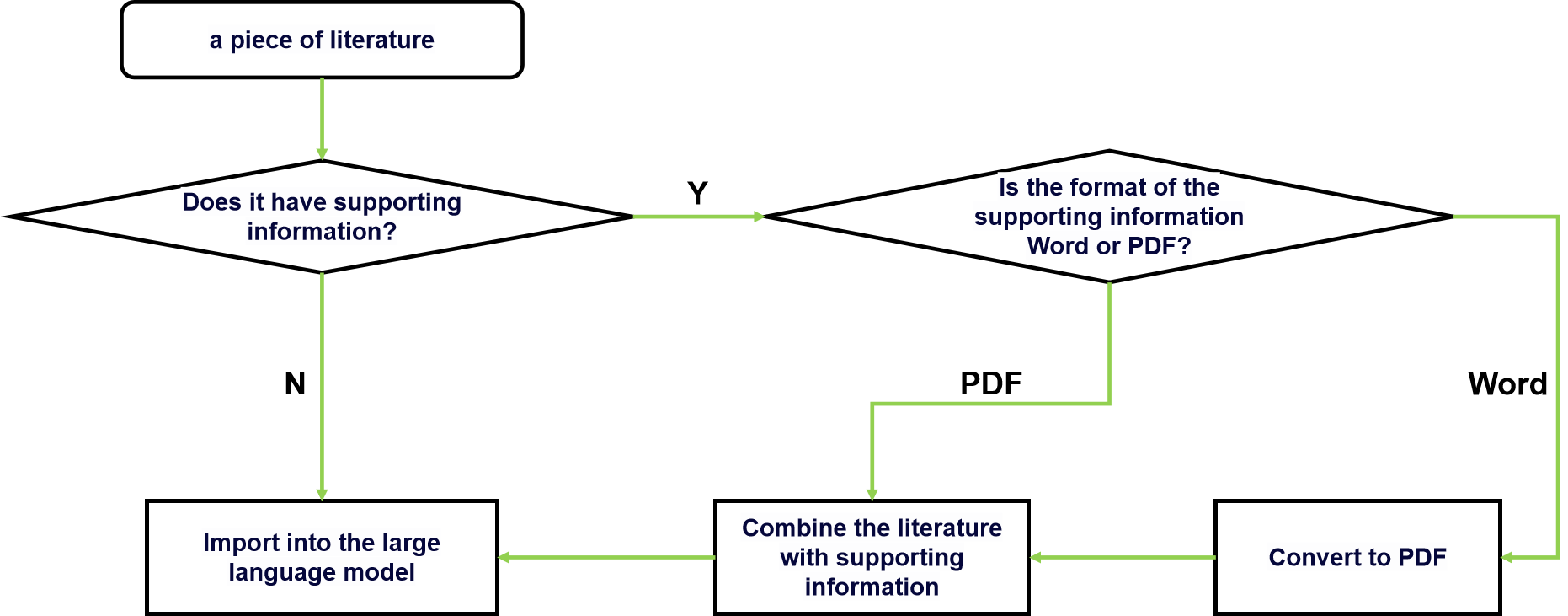


**Figure S2.** Diagram of Literature Preprocessing. For papers without supporting information, no further processing is required. For those with supporting information, if the supplementary file is in PDF format, it is directly merged with the main text. If it is in Word format, the file is first converted to PDF and then merged with the main text.


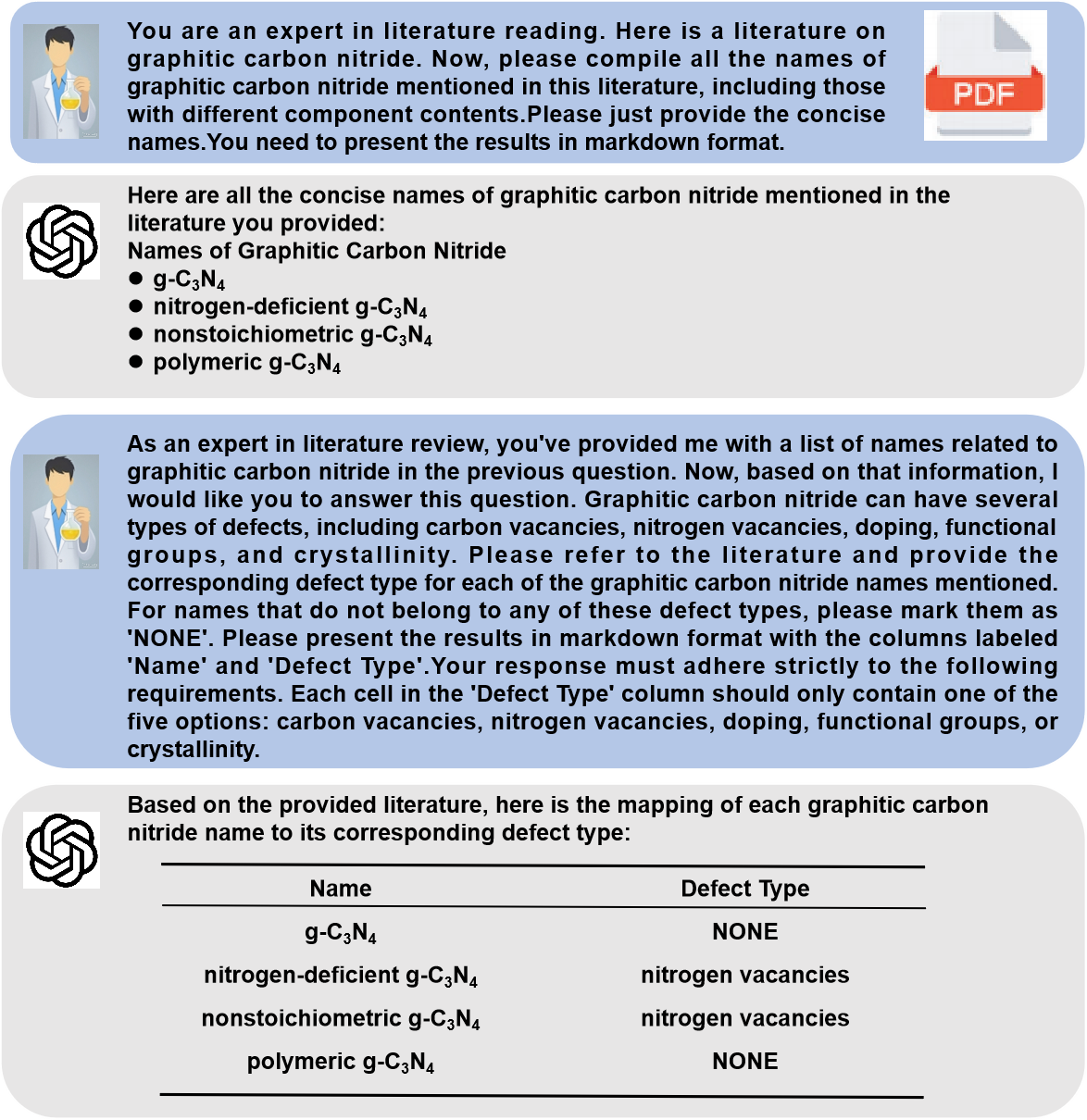


**Figure S3.** Dialogue example: Interactive question–answering for literature information extraction on g-C_3_N_4_ using GPT-4o.


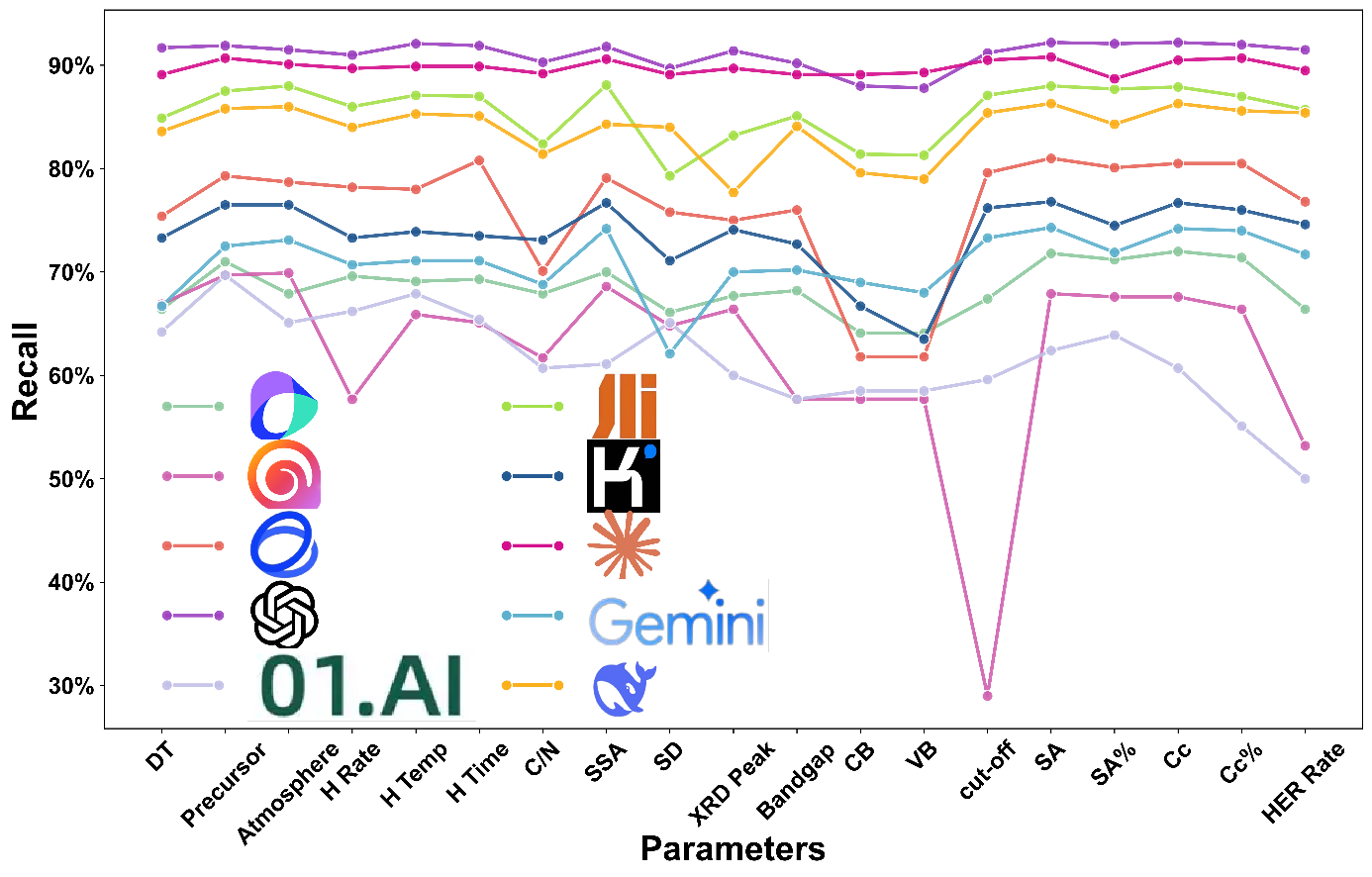


**Figure S4.** Line chart of recall for the extraction of different parameters by ten LLMs.


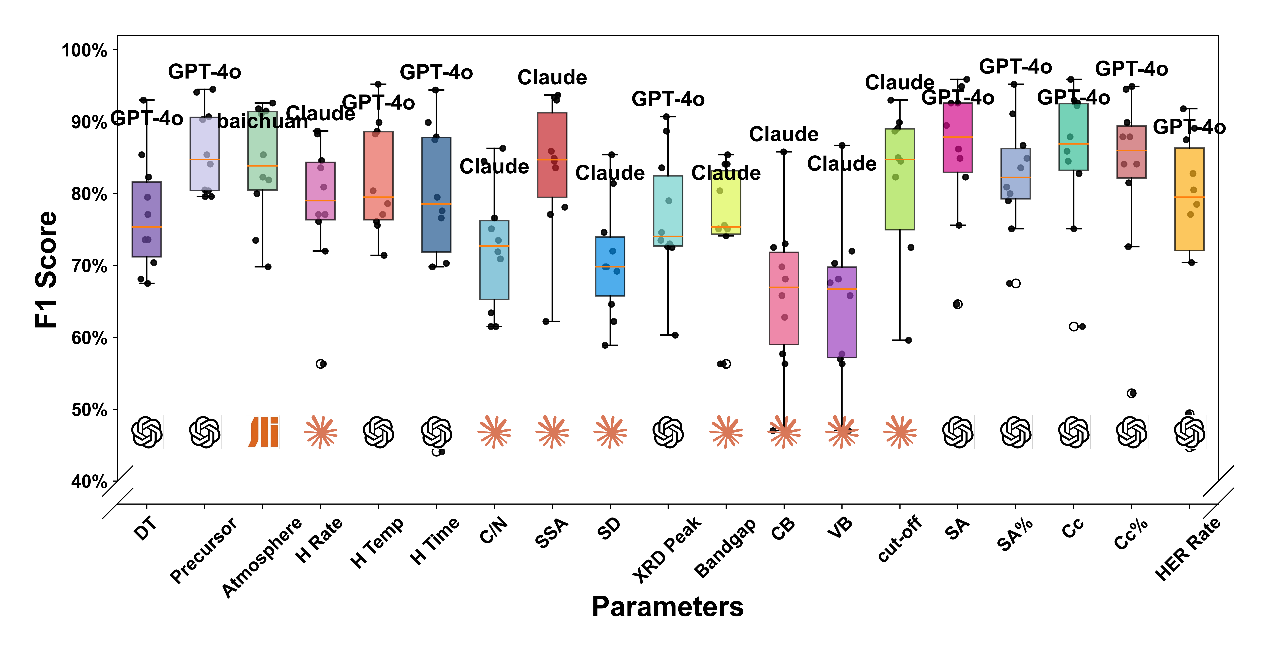


**Figure S5.** Box plots of F1 score for the extraction of different parameters by ten LLMs.


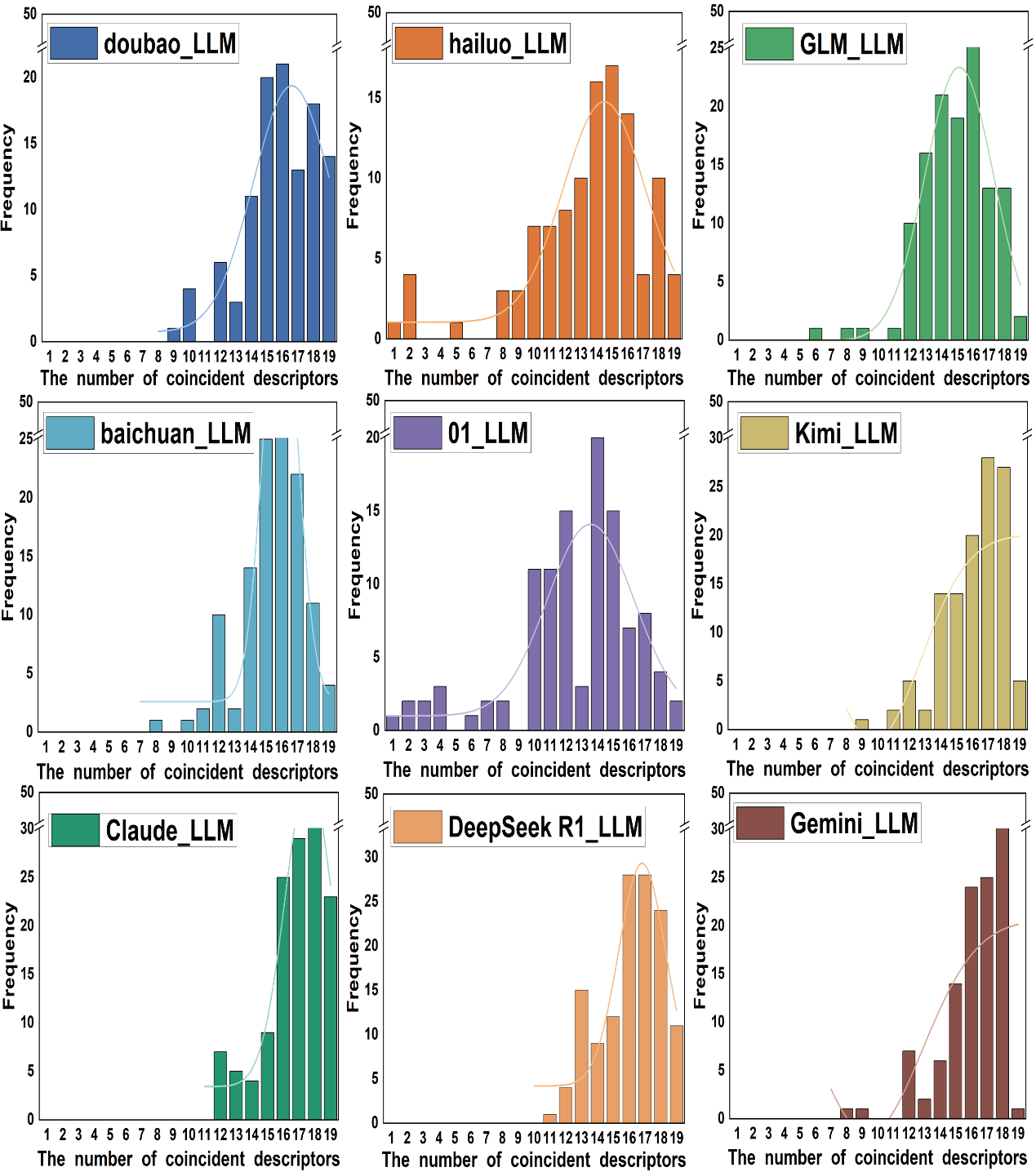


**Figure S6.** Histogram of the frequency distribution for a single model. The x-axis indicates the number of parameters (out of 19) correctly extracted by the model for a single data entry. As a comparison to GPT-4, the frequency distribution histogram of GPT-4o is shown in Figure S7.


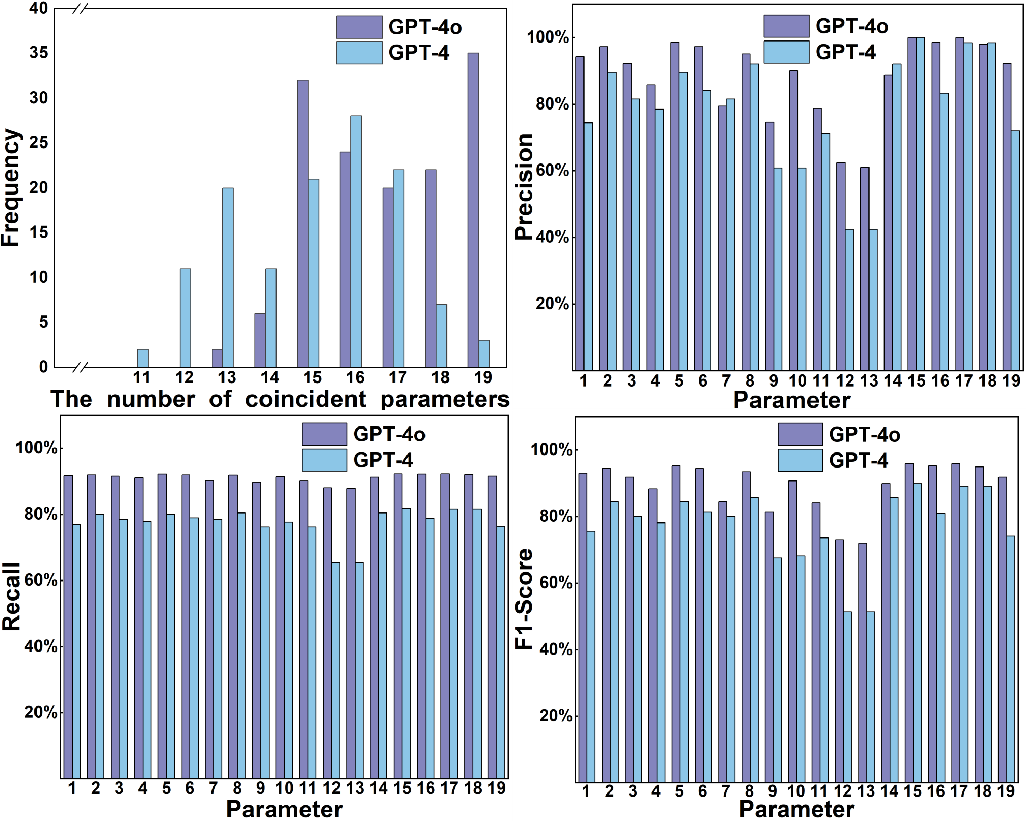


**Figure S7.** Comparative analysis of GPT-4o and GPT-4 across four evaluation metrics: frequency distribution (per data entry), precision, recall, and F1 score.


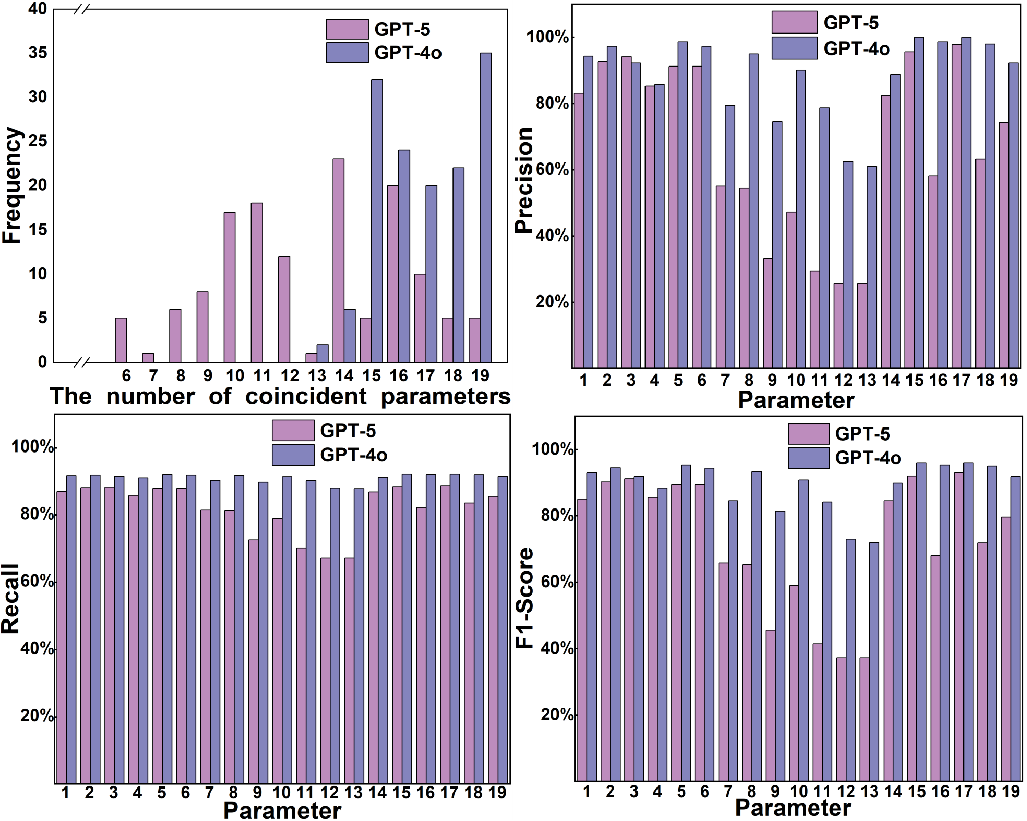


**Figure S8.** Comparative analysis of GPT-4o and GPT-5 across four evaluation metrics: frequency distribution (per data entry), precision, recall, and F1 score.


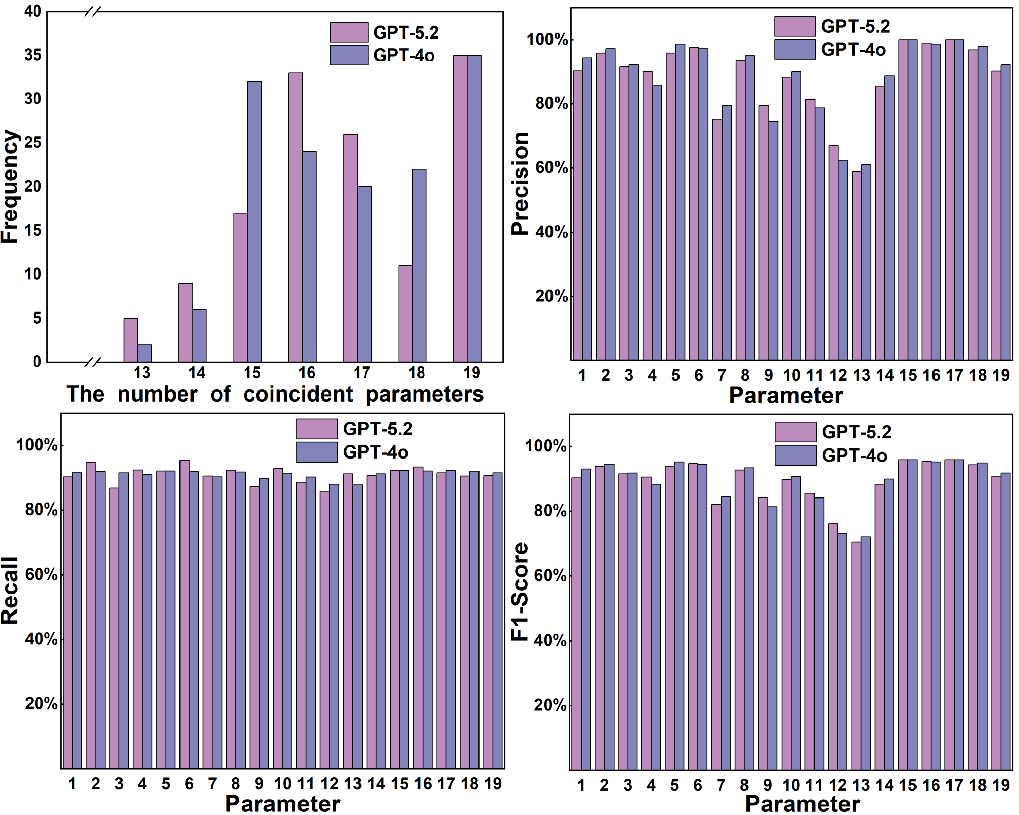


**Figure S9.** Comparative analysis of GPT-4o and GPT-5.2 across four evaluation metrics: frequency distribution (per data entry), precision, recall, and F1 score.


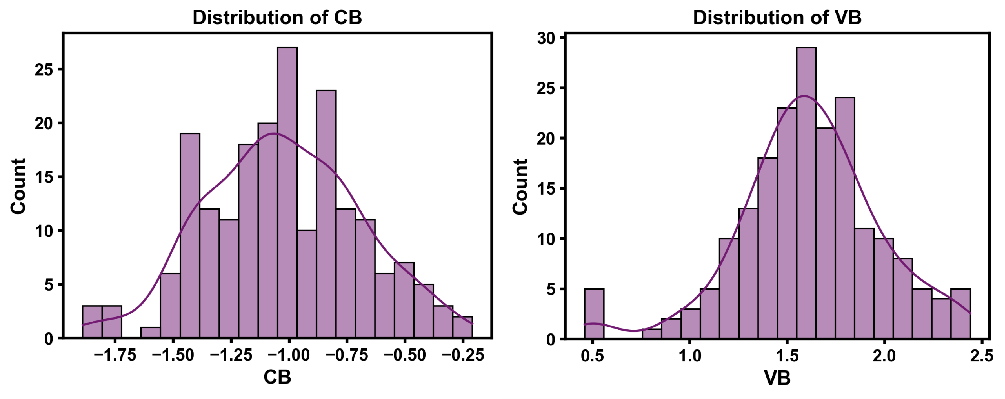


**Figure S10.** Distribution of CB and VB.


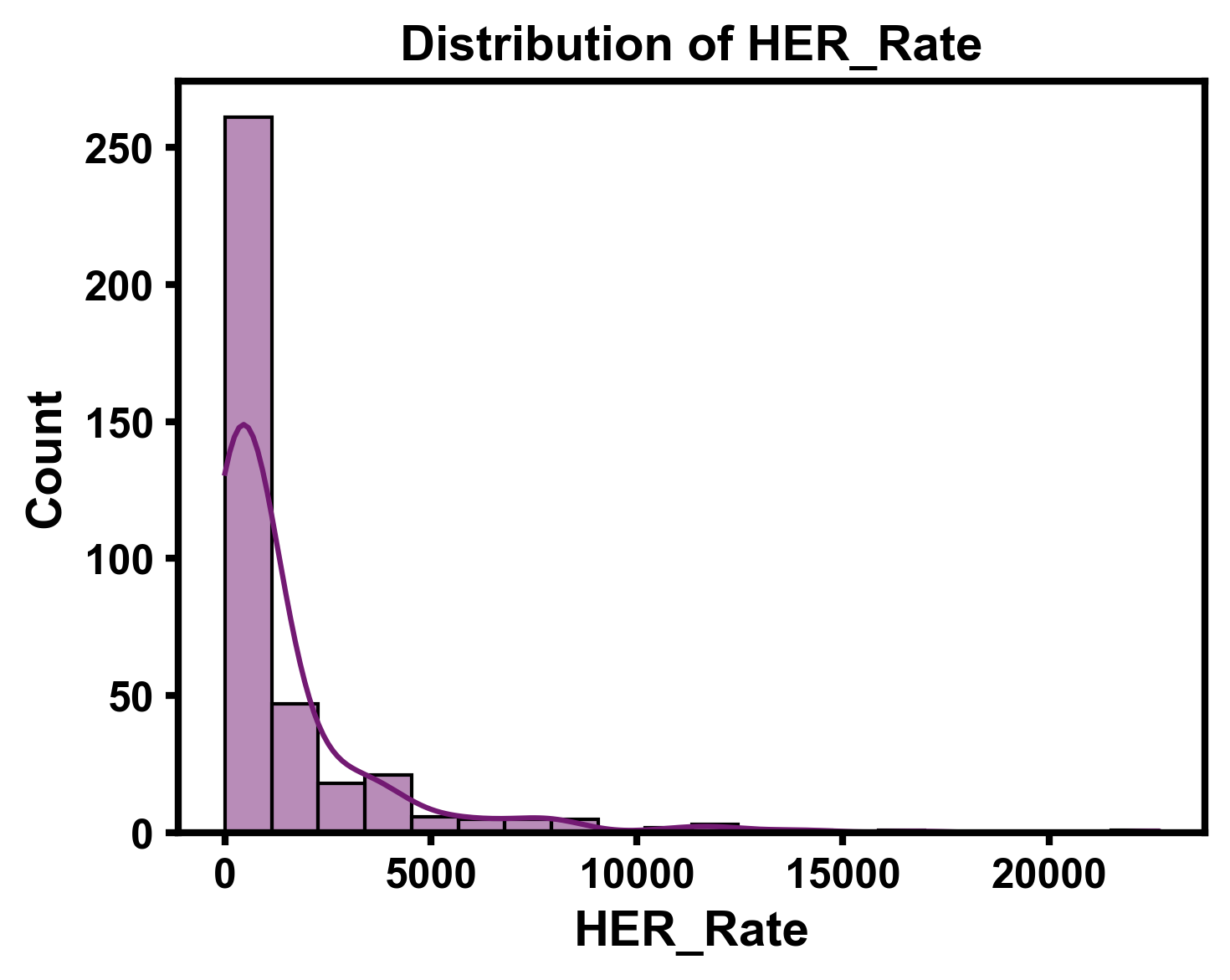

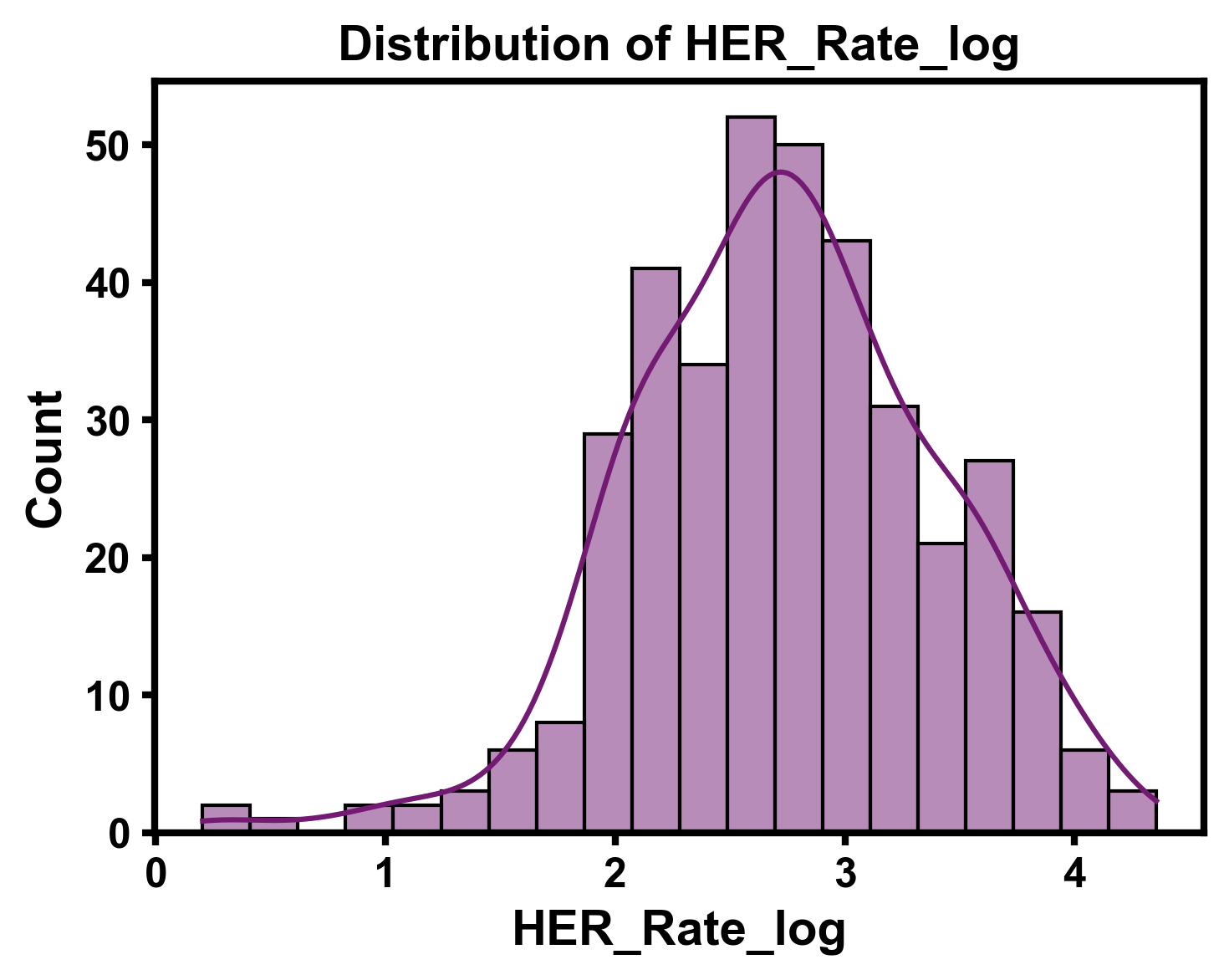


**Figure S11.** Distribution of the target variable before and after log transformation.


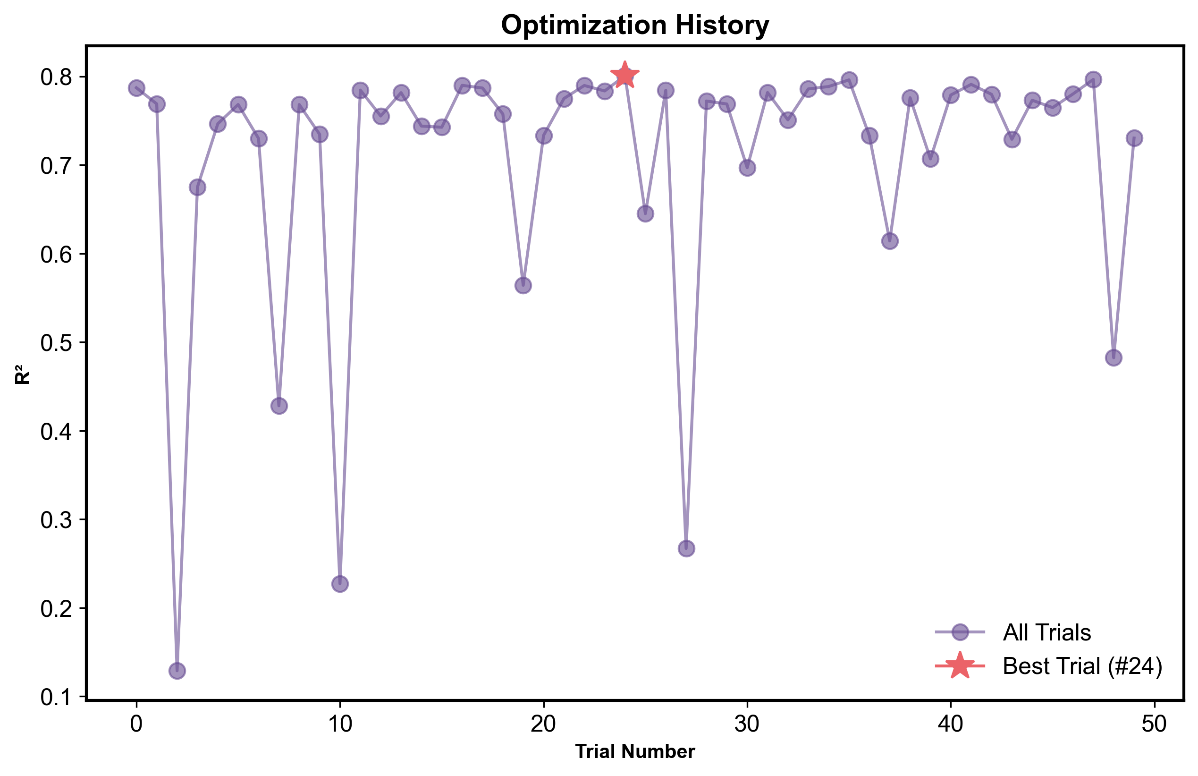


**Figure S12.** Hyperparameter optimization history of the CatBoost model using Optuna (R² score over trials).


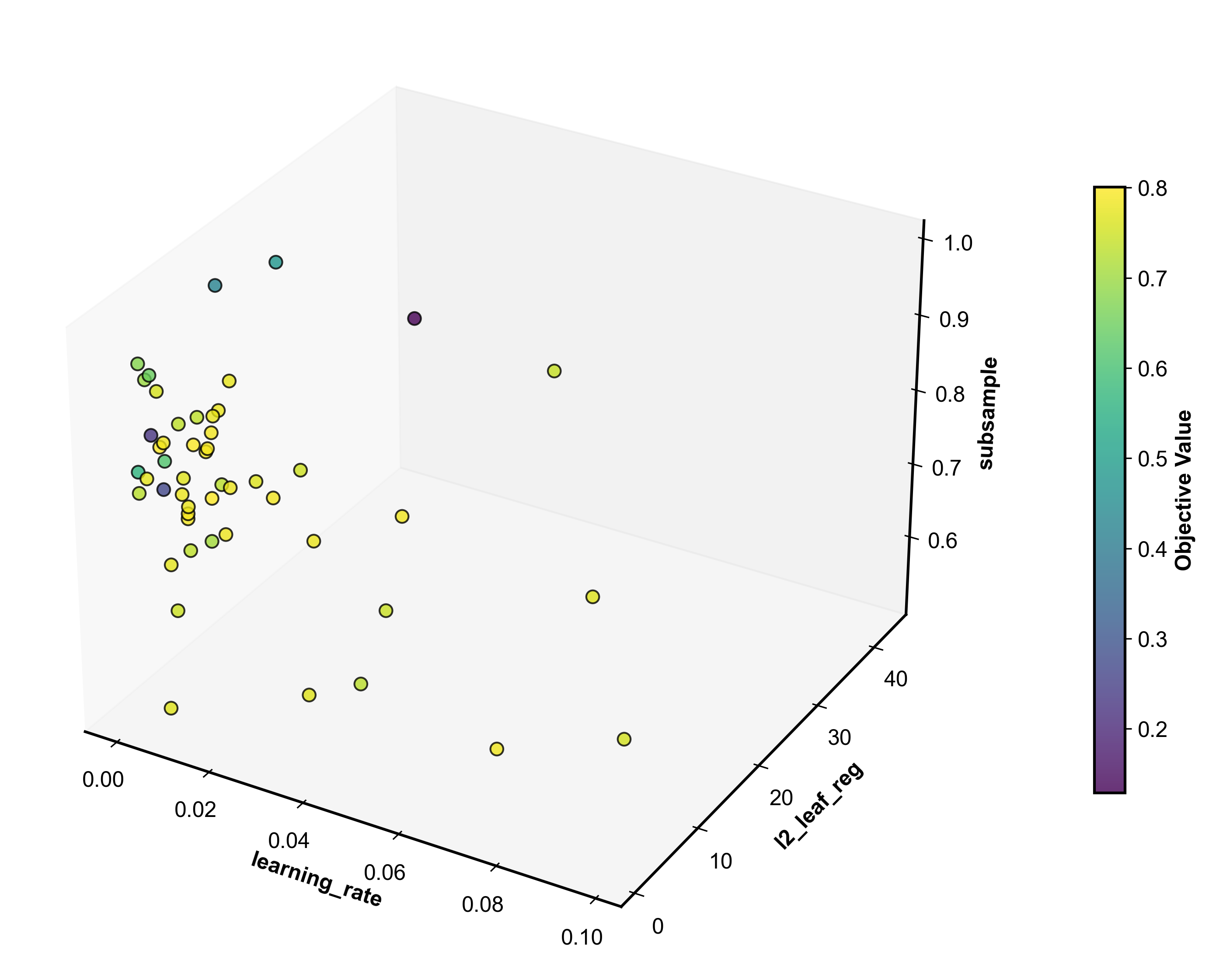


**Figure S13.** Three-dimensional visualization of the CatBoost hyperparameter optimization landscape using Optuna (Objective Value vs. learning_rate, l2_leaf_reg, and subsample).


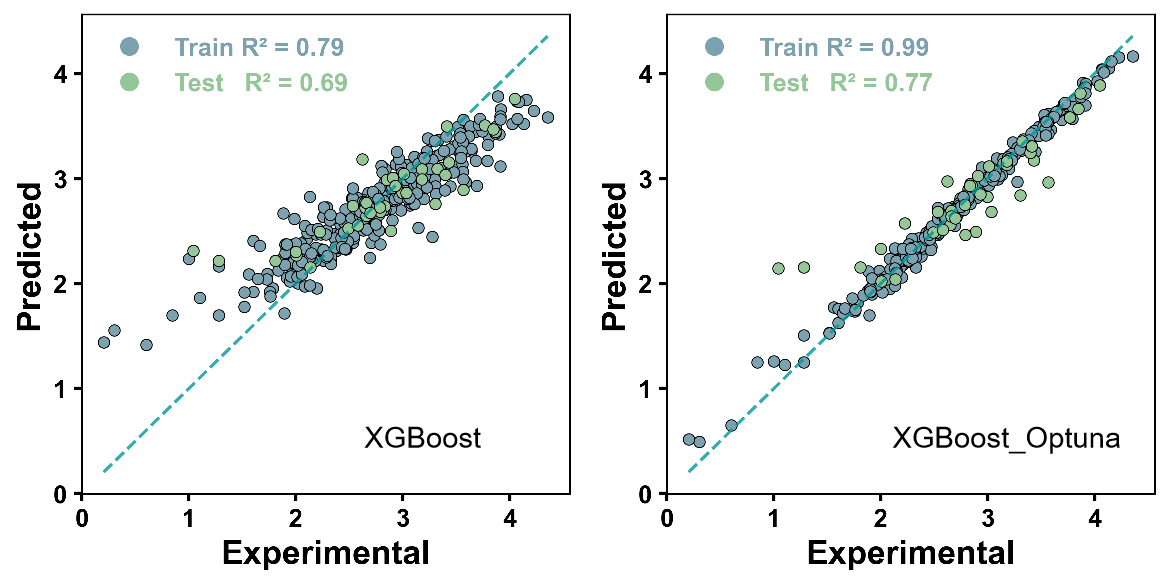


**Figure S14.** Comparison of predicted vs. experimental values for the XGBoost model before and after hyperparameter optimization using Optuna.


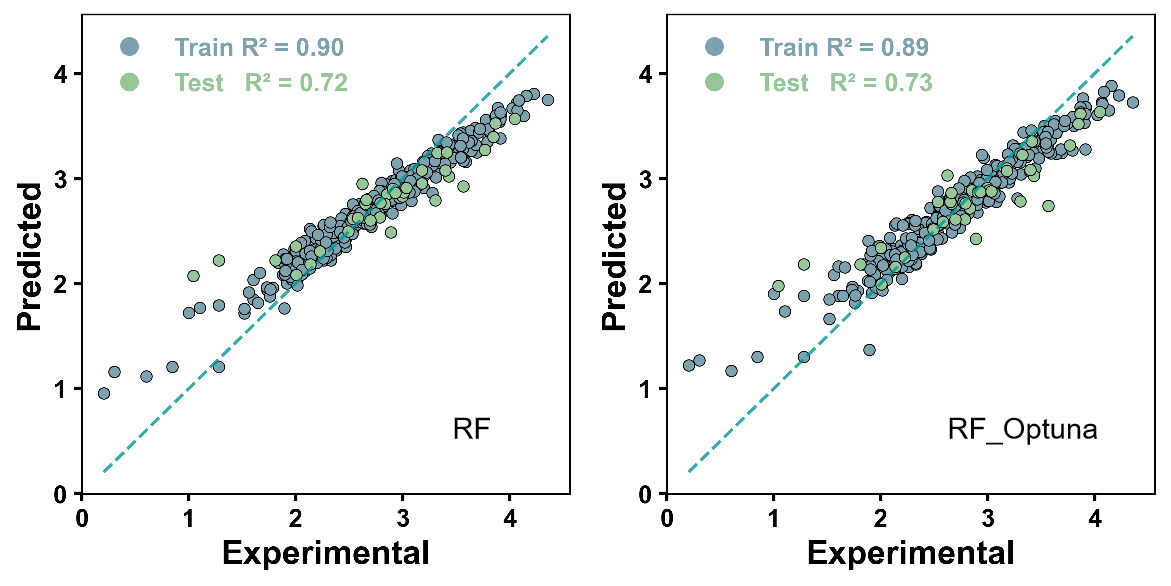


**Figure S15.** Comparison of predicted vs. experimental values for the RF model before and after hyperparameter optimization using Optuna.


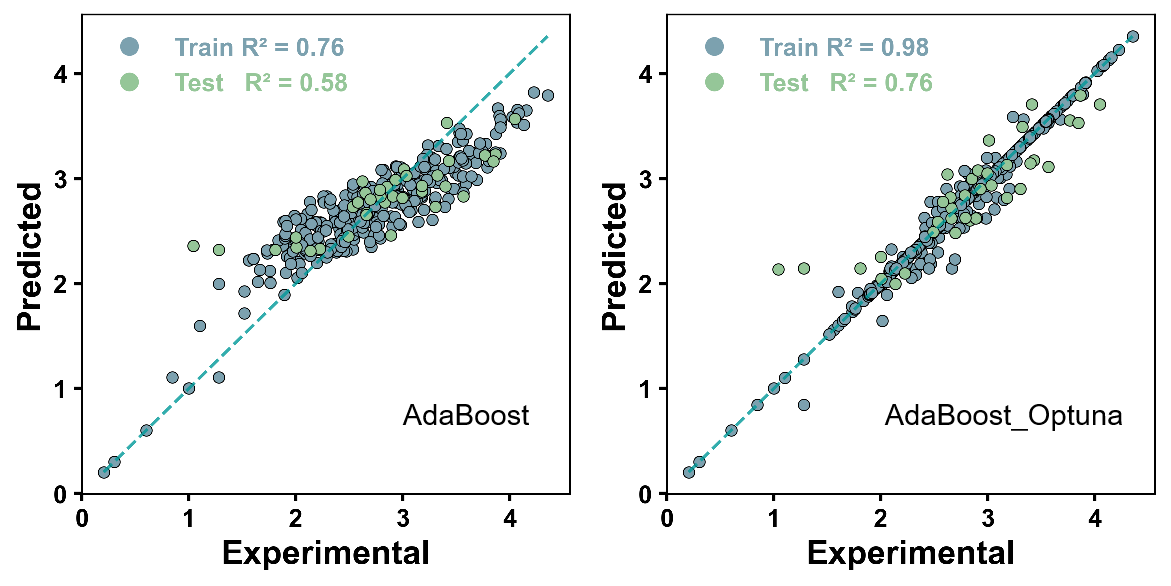


**Figure S16.** Comparison of predicted vs. experimental values for the AdaBoost model before and after hyperparameter optimization using Optuna.


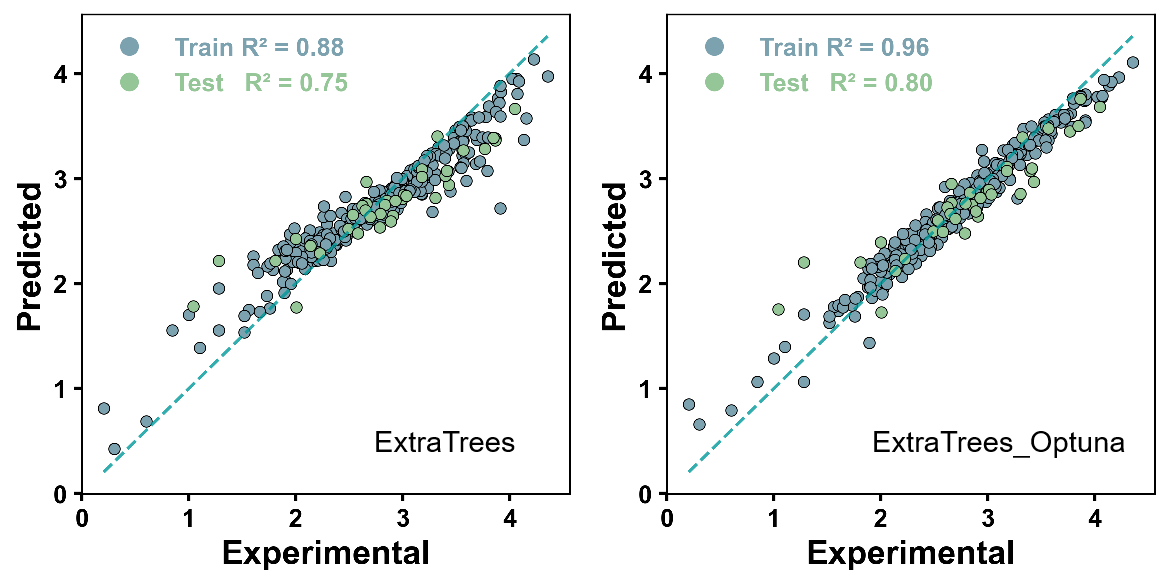


**Figure S17.** Comparison of predicted vs. experimental values for the ExtraTrees model before and after hyperparameter optimization using Optuna.


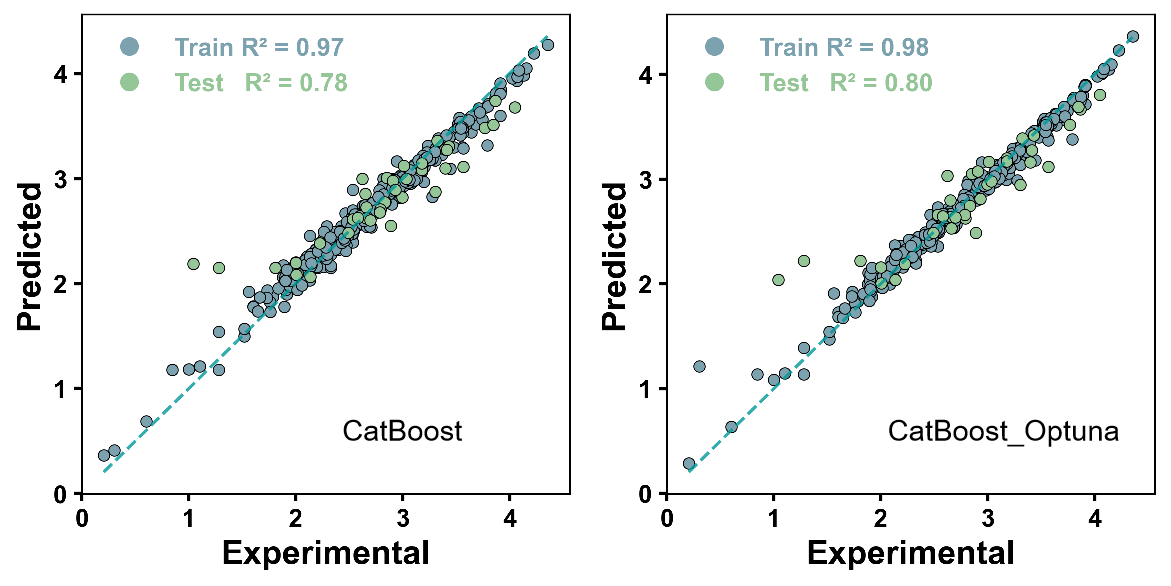


**Figure S18.** Comparison of predicted vs. experimental values for the CatBoost model before and after hyperparameter optimization using Optuna.


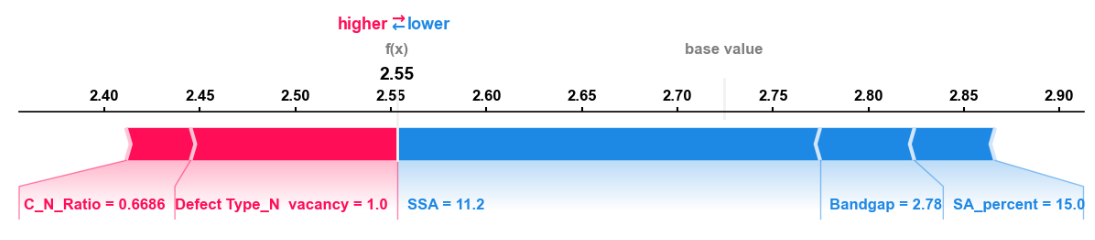


**Figure S19.** SHAP force plot illustrating feature contributions to the CatBoost model prediction for a representative sample.


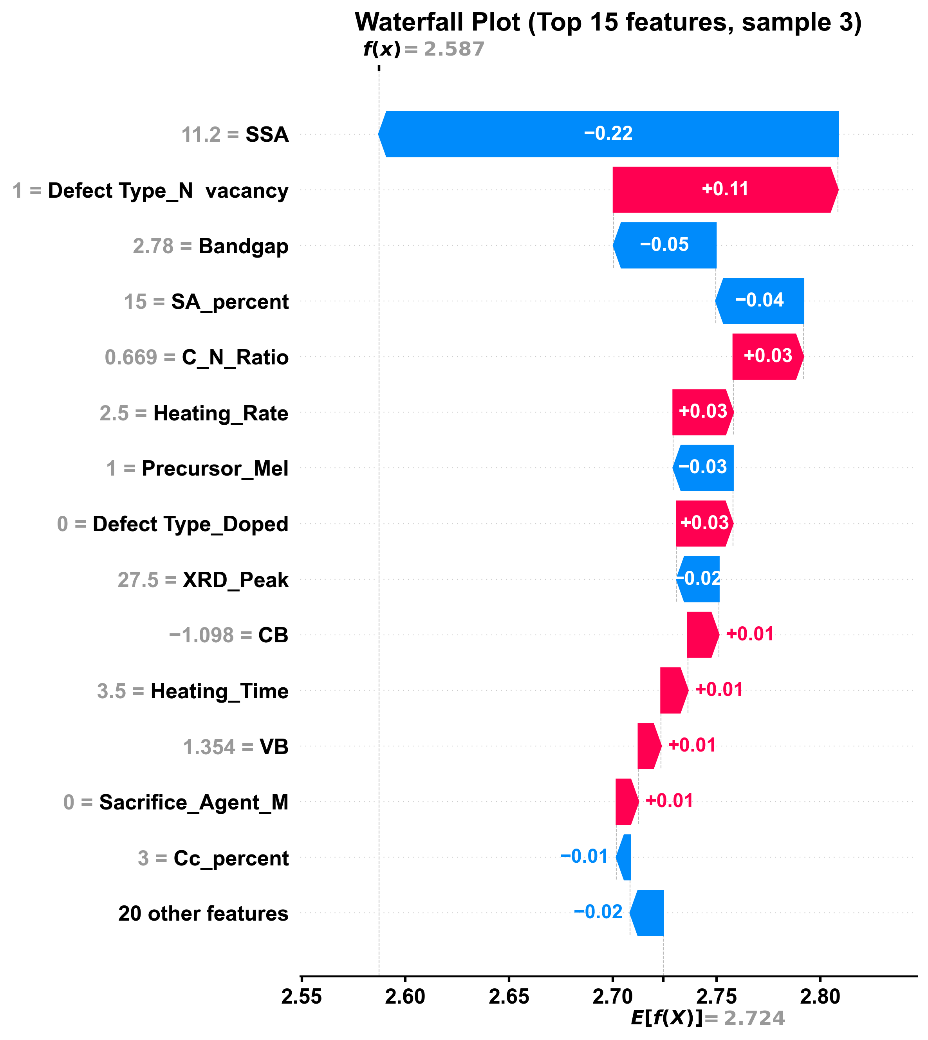


**Figure S20.** SHAP waterfall plot illustrating the cumulative contributions of the top 15 features to the CatBoost model prediction for a representative sample.


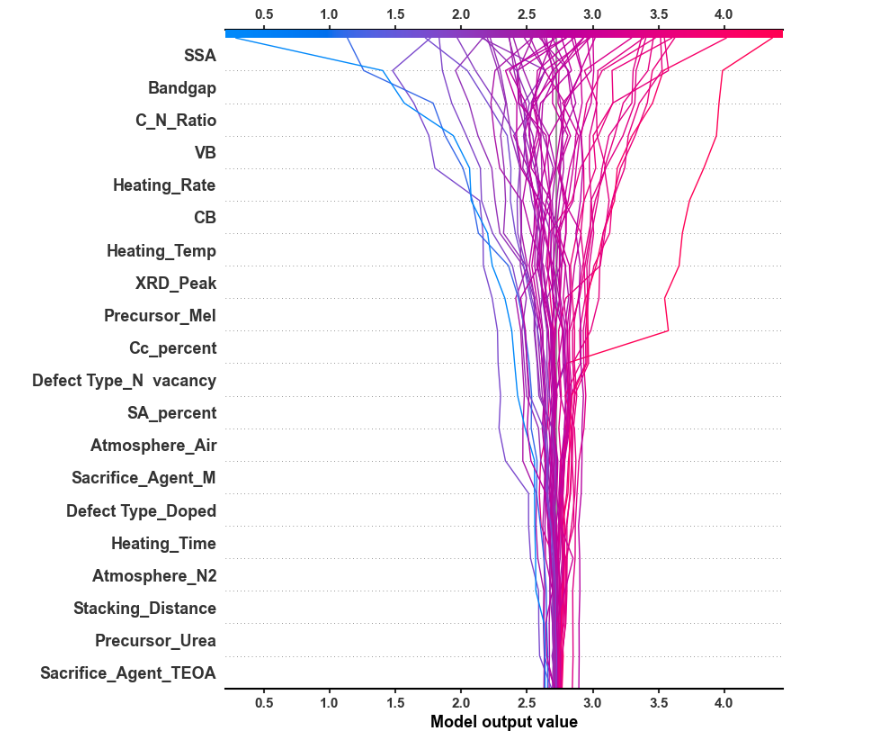


**Figure S21.** SHAP decision plot illustrating feature contributions across 50 samples in the CatBoost model.


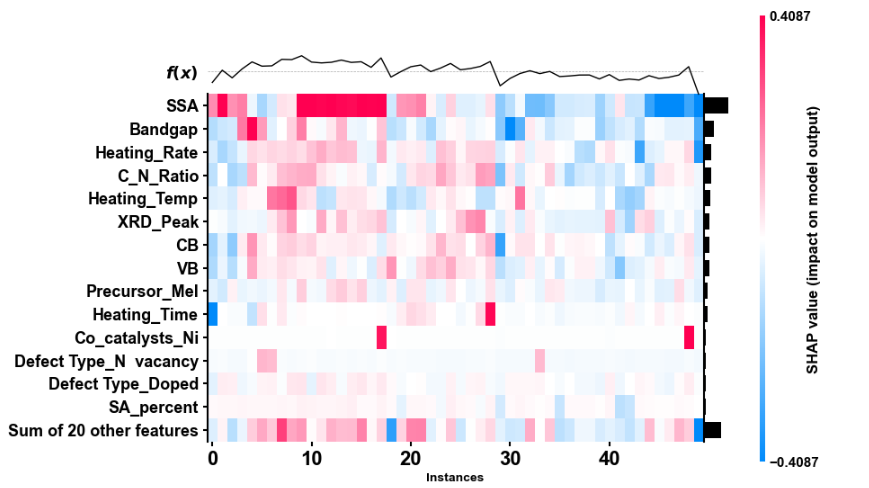


**Figure S22.** SHAP heatmap illustrating feature contributions to the CatBoost model predictions across 50 samples.


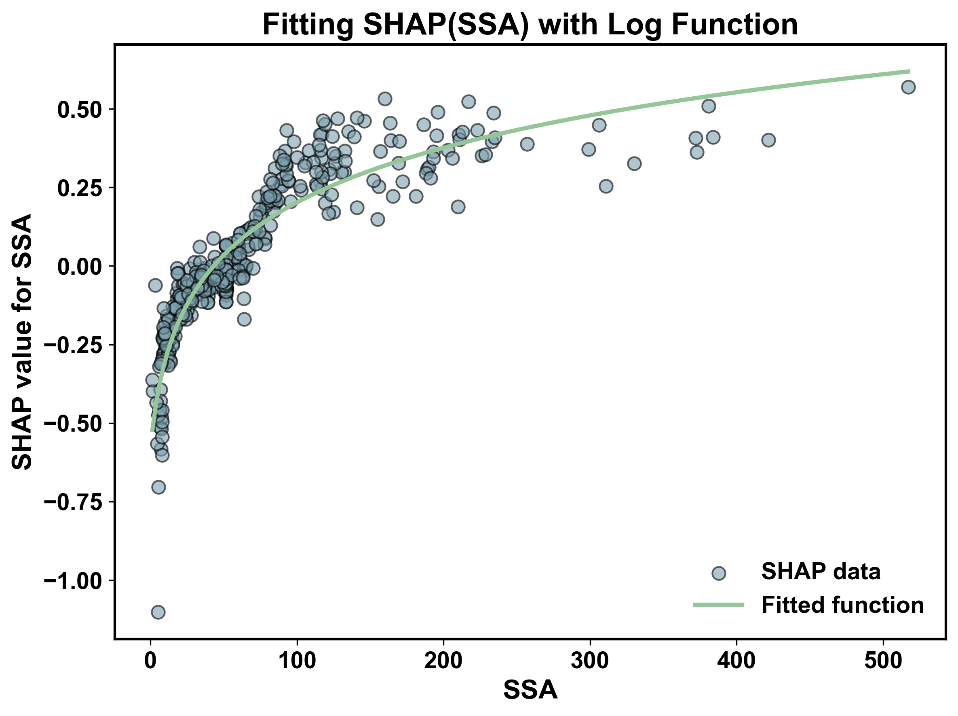


**Figure S23.** Fitted relationship between SHAP values and SSA feature using a logarithmic function.


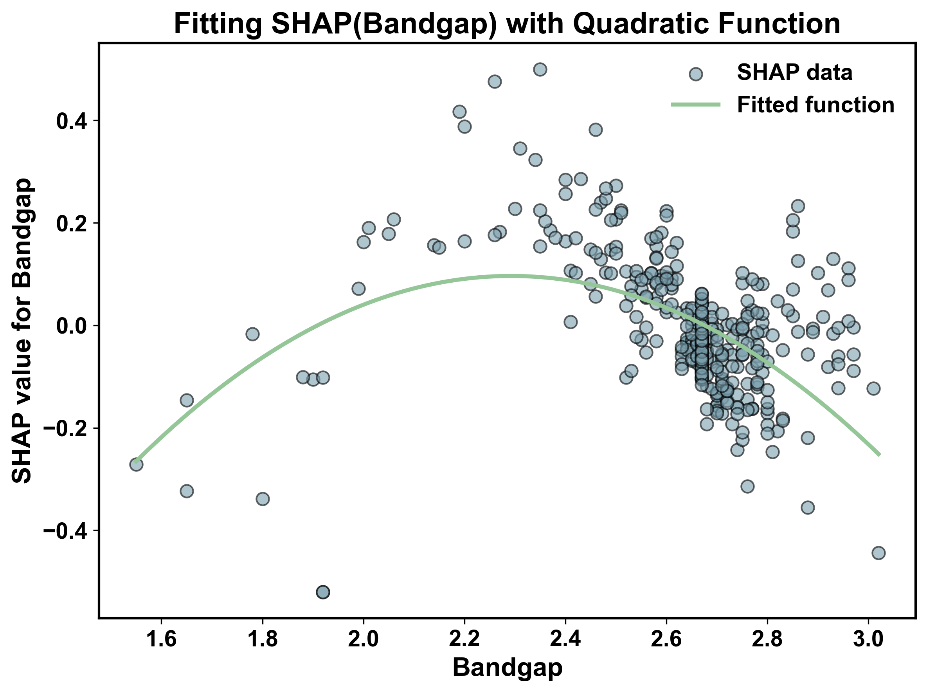


**Figure S24.** Fitted relationship between SHAP values and Bandgap using a quadratic function.


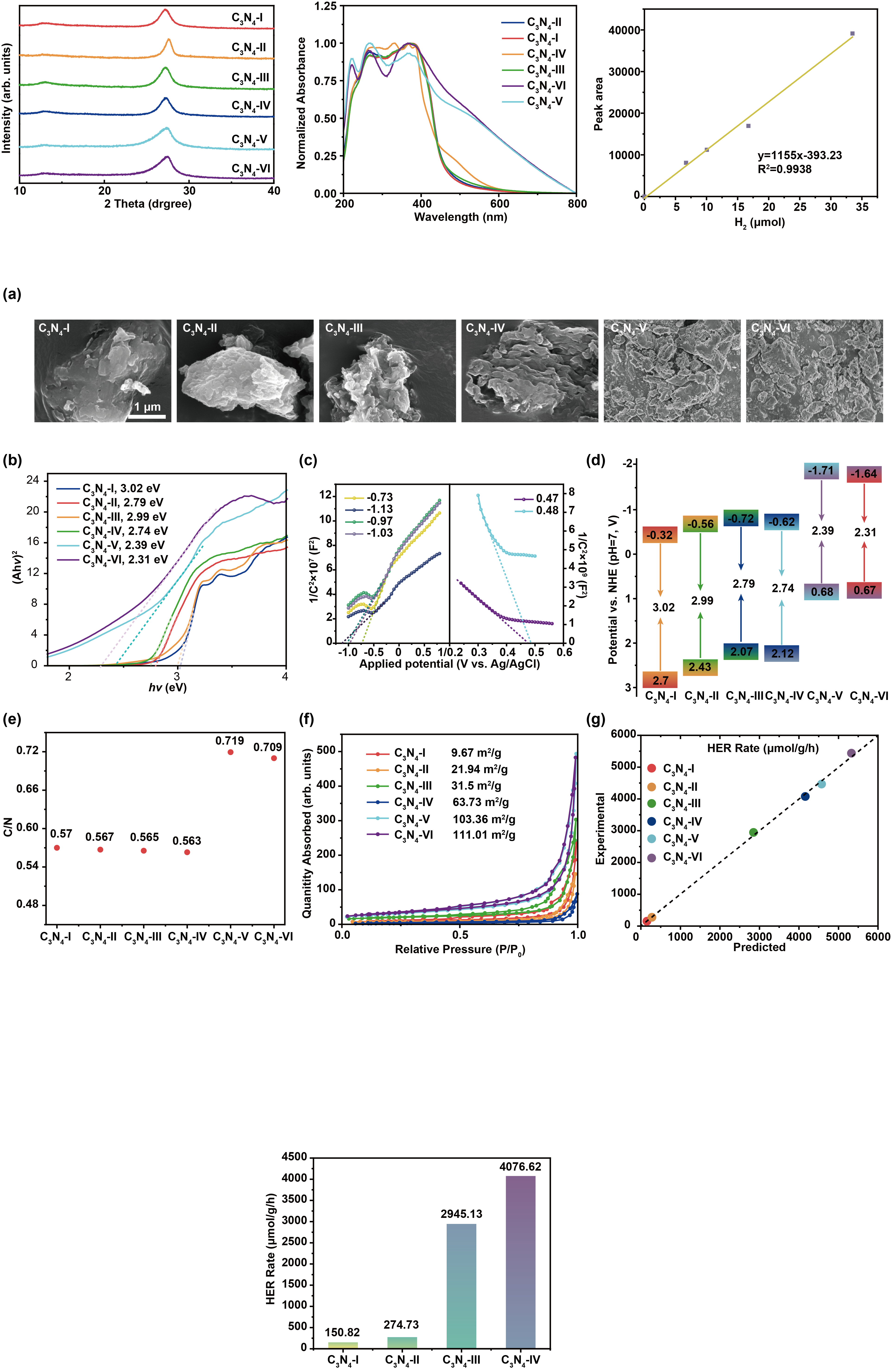


**Figure S25.** The XRD patterns of different g-C_3_N_4_.


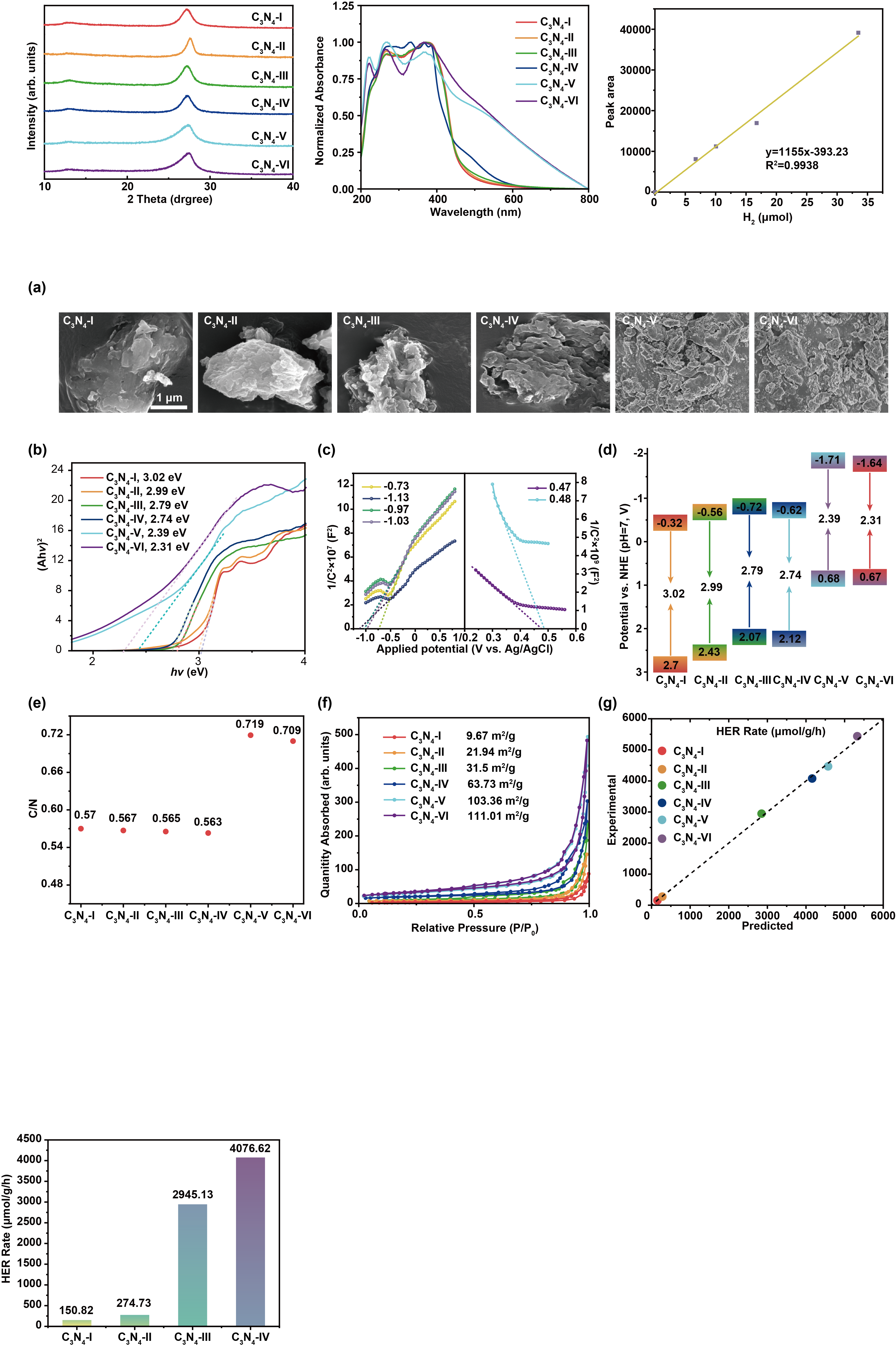


**Figure S26.** The UV–vis spectra of different g-C_3_N_4_.


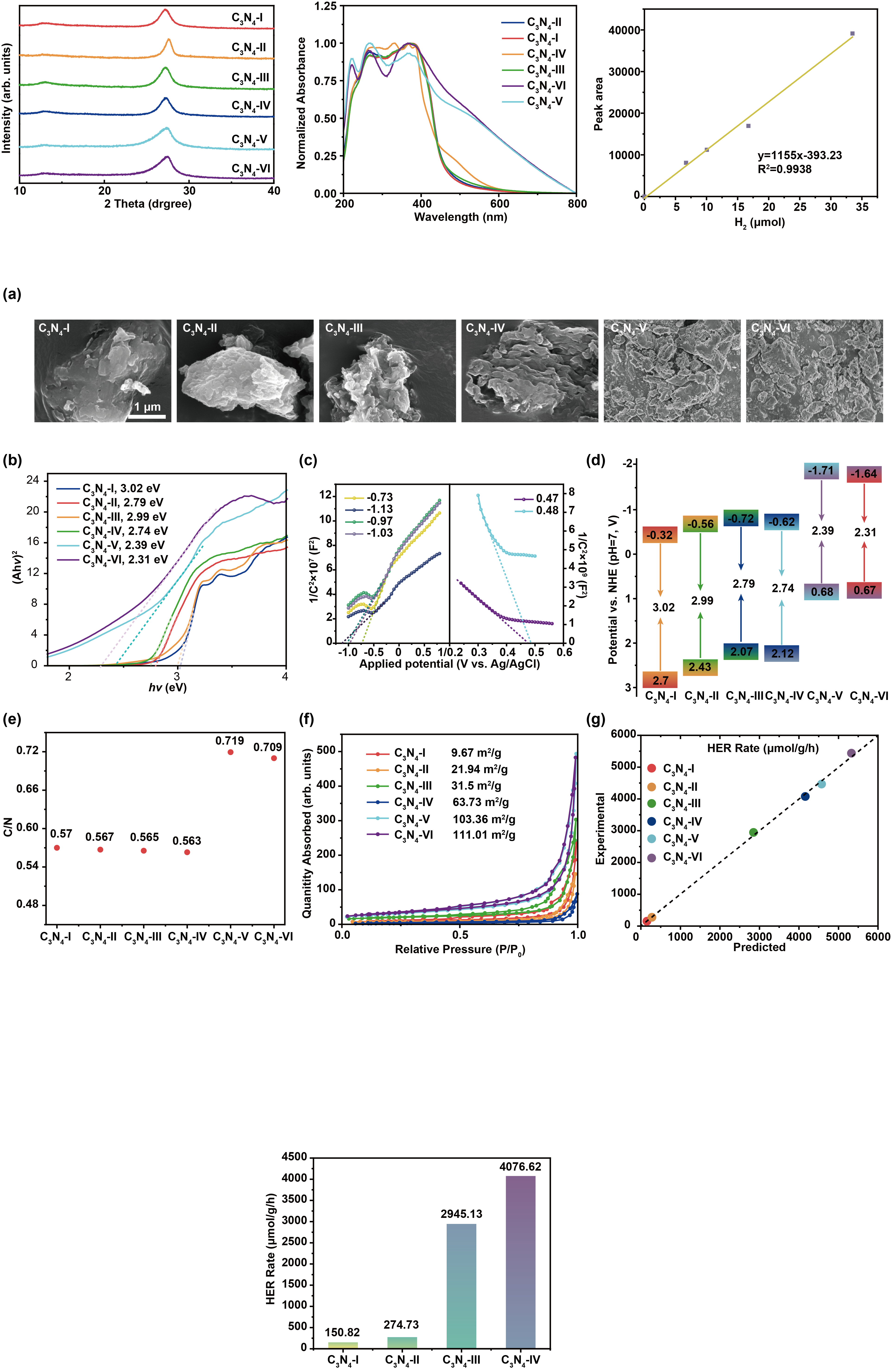


**Figure S27.** Standard curve of peak area versus hydrogen concentration.
